# Genotypic variability of Tunisian maize landraces: A valuable genetic resource to mitigate drought and heat stress in the Mediterranean basin

**DOI:** 10.1371/journal.pone.0338577

**Published:** 2025-12-16

**Authors:** Mohamed Dhia Eddine Hammami, Zayneb Kthiri, Walid Hamada, Rosa Ana Malvar, Chahine Karmous, Pedro Revilla

**Affiliations:** 1 Laboratory of genetics and cereals breeding, National Institute of Agronomy of Tunisia, Carthage University, Tunisia; 2 Department of Plant Biology and Soil Science, Faculty of Biology, Universidade de Vigo, Spain; 3 Misión Biológica de Galicia (Spanish National Research Council, CSIC), Pontevedra, Spain; KGUT: Graduate University of Advanced Technology, IRAN, ISLAMIC REPUBLIC OF

## Abstract

This study aimed to assess the drought and heat stress tolerance of nine Tunisian maize populations and their potential stress tolerance mechanisms. Over two years, nine Tunisian maize populations were evaluated under five environments with varying stress levels and one optimal growth condition in Tunisia. This work formed part of a larger study that includes a total of 223 Mediterranean maize landraces. The nine Tunisian populations were specifically chosen to assess the behavior of landraces adapted to the drought and heat stress conditions prevalent in the southern Mediterranean. In all the locations, the trials followed an augmented design with five blocks and a total of five checks over the two-year study period replicated in each block.The study demonstrated that combined drought and heat stress severely reduced maize yield, with Tunisian landraces experiencing losses of 76% to 95% relative to optimal conditions. Factorial regression analyses were performed to provide a biological interpretation of the contribution of environmental and genotypic variables, as well as their interactions, to grain yield variability. The most representative genotypic covariates were plant height (PH) followed by the number of ears (NE), thousand-grain weight (1000GW), and aerial biomass, respectively, explaining 26%, 12%, 9%, and 8% of the total variability. The significant environmental covariates were cumulative hydric deficit (DHC) and the average anthesis silking interval (ASI_ENV) in each environment, representing 48% of the total environmental variation. The interaction between thousand-grain weight and cumulative hydric deficit had the highest contribution (9%) of interaction for grain yield. The factorial regression indicated that under stress conditions, maize plants appeared to adapt to maintain yield by increasing thousand-grain weight while reducing aerial biomass, number of ears, and grain number. This response likely reflects an enhanced capacity for efficient resource reallocation, supporting the plant’s resilience under combined drought and heat stress conditions. The landraces BK, KAR, and MT2 consistently outperformed in most traits under stress conditions, showing significant tolerance and adaptability for across multiple stress levels with better yields and flowering synchronization. The selected best-performing populations could serve as valuable sources of drought and heat stress tolerance sources for future breeding programs.

## Introduction

Maize (*Zea mays* L.) is the most important cereal crop after rice and wheat and is cultivated worldwide due to its importance as a primary food resource, livestock feed, and biofuel [1,2]. Maize production, particularly in the Mediterranean areas, faces many challenges, such as water and heat stress (Rida et al., 2021). Maize is more heat and drought-susceptible than rice (*Oryza sativa* L.) and wheat (*Triticum aestivum* L.), mainly when high temperatures and drought occur during flowering and grain-filling growth stages [3,4]. Drought and heat stress are expected to rise in all projected climate change scenarios thus limiting crop growth and productivity [5,6].

Maize plants under drought stress experience reductions in photosynthetic rate [7], growth, yield, and grain quality, particularly through a decline in starch relative to protein content [8,9]. Depending on its intensity and duration, drought causes maize yield loss of 30% to 90% [10,11]. Heat stress alone also severely reduces maize yield; each increase of one degree Celsius exceeding 30°C reduces maize grain yield by 1% to 1.7% [12]. Global warming scenarios expect a reduction of maize yield from 21% to 50% under 1°C to 4°C increase, respectively [13]. The favorable growth temperature for maize is 26°C, and for each degree increase of this temperature, root and shoot mass both decline by 10% until 35°C when growth is the most retarded [14].

Like other crops, maize performance varies considerably across environments due to Genotypes × Environments (G × E) interactions [15]. The interaction between Genotypes and Environments suggests that genotype ranking is inconsistent across several environments [16]. This differential performance can be classified into two main interaction; a Magnitude interaction and a crossover interaction [17]. Such interactions are critical in plant breeding programs, as they influence selection efficiency and the stability of genotypic performance across diverse growth conditions [17]. Therefore, under various environmental conditions, yield could be affected by the effect of the environment (E), the genotype (G), and their interaction (G × E). Assessing G × E is thus critical for breeding programs, either to select broadly adapted genotypes with stable performance or to identify varieties specifically adapted to particular agro-ecological conditions [18]. Multi-environment trials (METs) and associated biometrical tools are essential to capture these dynamics and guide breeding strategies [19–22]. Despite advances in maize improvement, relatively few studies have addressed the combined effects of drought and heat stress under Mediterranean conditions, where these stresses frequently co-occur and severely limit yield stability. Moreover, the potential of local landraces as a genetic resource for stress tolerance remains underexploited.

The present study aims to (i) identify tolerant Tunisian maize local landraces under six contrasting environmental conditions, (ii) evaluate the G × E interaction of the Tunisian maize landraces across stressed and non-stressed conditions in order to identify high-performing genotypes and (iii) assess the relative impact of environmental, genotypic, and G × E interaction factors on maize grain yield, and investigate stress tolerance mechanisms and resource allocation under limited conditions.

## Materials and methods

### Plant material

We tested nine maize local landraces representing the unique Tunisian germplasm pools that have been adapted by selection to the Mediterranean climatic conditions and came from crosses and recombination of European and American sources [23,24] (Table 1). A total of five checks were used during the two-year study, composed of three Tunisian commercial hybrids (Agrister, Sancia, Fandari), the synthetic BS17, and the hybrid B73 × Mo17. Agrister, Fandari and Sancia were the commercial Tunisian hybrids used as checks during the first year of the study [25]. The synthetic BS17 and the hybrid B73 × Mo17 were also used as checks, accompanied by Agrister and Fandari during the second year of trials.

**Table 1:** Tunisian maize landraces evaluated in this study, along with the regions and bioclimatic zones.

Landrace_ID	Region	Latitude	Longitude	Bioclimatic zone
BIZ1	Bizerte	37°18’10.7“N	9°49’57.0“E	Subhumid
BIZ2	Bizerte	37° 18’ 58.5“N	9° 41’ 57.7“E	Subhumid
GAB1	Gabès	33°52’39.3“N	10°03’53.0“E	Semi-arid with cold winter
GAB2	Gabès	33° 51’ 4.37“N	10° 7’ 22.76“E	Semi-arid with cold winter
GAF	Gafsa	34°25’49.9“N	8°46’05.9“E	Arid with cold winter
KAR	Kairouan	35°36’57.0“N	9°55’23.2“E	Semi-arid with cold winter
MT1	Nabeul	36°47’11.7“N	10°59’52.2“E	Subhumid
MT2	Nabeul	36°55’48.0“N	11°06’00.0“E	Subhumid
BK	Nabeul	36°38’54.1“N	10°35’50.4“E	Subhumid

### Field trials

This study focuses on nine Tunisian landraces, selected from a larger dataset of 255 Mediterranean varieties and selections cycles, in which only the Tunisian accessions were examined in detail for the specific objectives of the present work. The field trials followed an augmented design with a total of five checks over the two-year study period. In the first year, the design included 5 blocks with 54 plots per block. Of these, 3 plots in each block were allocated to check varieties, which were replicated across all blocks, while the remaining 51 plots were assigned to unreplicated landraces. In the second year, the augmented design consisted of 55 plots per block, with 4 plots assigned to replicated check varieties and 51 plots allocated to unreplicated landraces.

The nine Tunisian maize landraces were selected based on their diverse bioclimatic origins, which may confer adaptation to contrasting abiotic stresses. Previous assessments demonstrated substantial genetic diversity among them, indicating potential for differential tolerance [22,23]. This diversity represented a valuable resource for improving maize productivity under the challenging conditions of Tunisia and other Mediterranean regions with similar growth conditions. The genotypes including landraces and checks, were cultivated in a plot measuring three meters in length with 12 hills per row, each hill sown with two seeds. The interlinks between the experimental plots were 50 cm, and the plants on the same plot were spaced by 25 cm. Hills were thinned to one plant at two leaf stages, with an expected final plant density of around 60000 plants ha^-1^.

**Fig 1:**
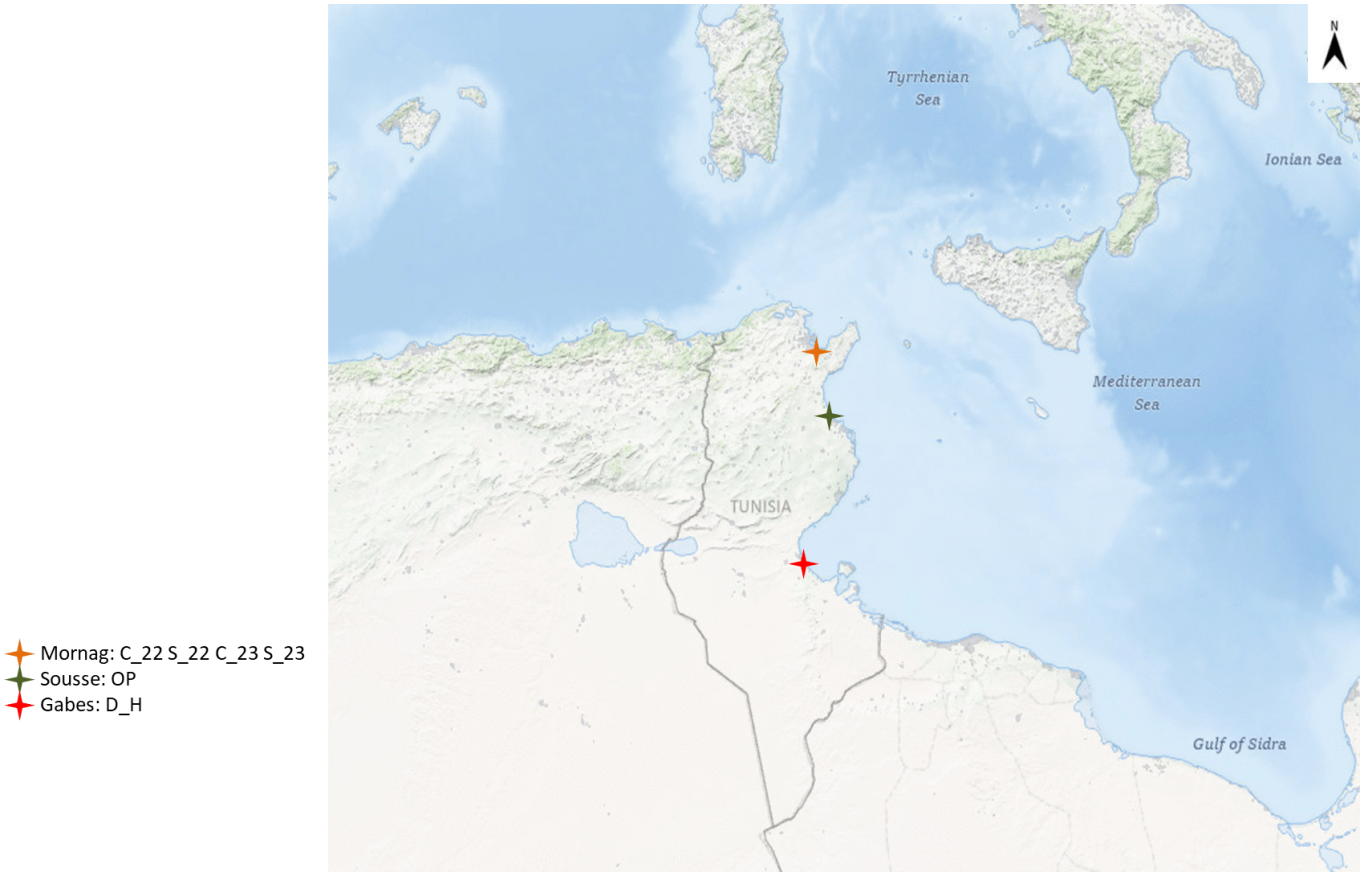
Experimental fields used for the screening trials of nine Tunisian maize landraces and check varieties across three locations in Tunisia. Mornag was used for control and stressed irrigation conditions in 2022 and 2023 (C_22, C_23, S_22, S_23), Sousse for optimal conditions (OP), and Gabès for combined drought and heat stress (D_H). Map of Tunisia generated using data from NASA Earth Observatory (public domain). Source: NASA Earth Observatory (https://earthobservatory.nasa.gov).

**Fig 2:**
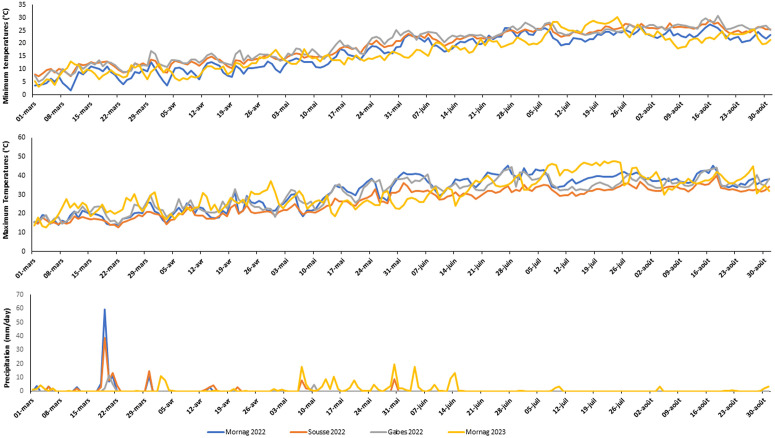
Precipitation, minimum, and maximum temperatures across the three Tunisian locations during maize growth cycle of the Tunisian maize landraces plus checks evaluated in 2022 and 2023.

The trials were conducted under six environments in three locations from different bioclimatic areas: Mornag, Sousse, and Gabèss (Fig 1). In Mornag, the soil texture was heavy clay with high organic content. In Sousse, the soil texture was sandy, while in Gabès, the soil has a sandy clay texture (Table 2). During the maize growth cycle in Mornag, temperatures ranged from 20°C to 45°C in 2022 and 2023. Several heat waves were recorded in Mornag during both years with more frequent heat waves in 2023 than in 2022, classifying it as a heat-stressed setting, even for the well-watered trials. The temperatures in Sousse during the growth cycle ranged between 20°C and 38°C, with no registered heat waves (Fig 2). The most stressful growth conditions were recorded in Gabès, with several heatwaves similar to those in Mornag, but more frequent during the initial growth stages (Fig 2). The heatwaves in Gabès were accompanied by a lack of precipitation with a total of only 15 mm during the cycle with low water input (Fig 2).

**Table 2:** Average main soil characteristics of the locations where the Tunisian maize landraces were evaluated: Mornag, Sousse and Gabès experimental fields at a depth of 60 cm.

Soil characteristics	Mornag	Sousse	Gabès
Clay (%)	47.0	22.2	13.9
Silt (%)	24.5	16.6	26.3
Sand (%)	28.5	61.2	59.8
Total limestone (%)	19.10	< 4.00	8.18
pH	8.07	8.01	8.19
Organic matter (%)	2.4	1.5	1.0
Electric Conductivity (mS/cm)	0.48	0.42	2.95
N total (‰)	1.76	0.96	0.61
P2O5 – assimilable (mg.kg-1)	21.93	35.69	4.68
K2O – assimilable (mg.kg-1)	745.2	521.3	294.3

Maize performance under different conditions was evaluated through two water regimes in Mornag with well-watered and stressed irrigation in 2022 and 2023, nominated as C_22 and C_23, respectively, for the control and S_22 and S_23 for the drought conditions. The control treatments of C_22 and C_23 received full irrigation at 90% of field capacity (FC), respectively, with a total water supply of 1507.3 mm and 1543.4 mm. The stressed environments received a moderate water deficit treatment for S_22 and S_23 by applying 50% of FC. The stress irrigation was applied approximately two weeks before tassel emergence. These water regimes were managed using the MABIA software integrated into the Water Evaluation and Planning (WEAP) framework. For both control and stress treatments, soil moisture was monitored using soil depletion curves, which tracked water availability in the root zone The total water inputs for S_22 and S_23 during the cycle were 1080.5 mm and 1112 mm, respectively. In Sousse (OP), the irrigation was applied regularly, four times weekly; the total water supply was 1610 mm during the cycle. In Gabès (D_H), irregular water availability disrupted the irrigation schedule, leading to irregular irrigation (once every 10–12 days), contributing to drought and heat stress conditions with frequent heat waves during the cycle. The total water supply was 710 mm. The irregular irrigation with the lack of precipitation and the high temperatures during the growth cycle, make Gabès the most stressed environment with a combined drought and heat throughout the entire cycle.

### Traits recorded

The number of days to pollen shedding (DT) and days to silking (DS) represented the number of days until 50% of the plants in the row showed tassels or silks, respectively. The anthesis-silking interval (ASI, days) was calculated as the difference between days to silking and days to pollen shedding. The morphological parameters such as plant height (PH, cm) and aerial biomass (AB, g) were recorded at harvest. Yield and yield-related parameters including the number of grains per ear (NG), number of grains per plant (NGP), thousand-grain weight (1000GW, g), ears per plant (NE), and grain yield per plant (GYP, g/plant) were also recorded on the same stage. The harvested ears were air-dried until reaching constant moisture before taking 1000GW and GYP. For the evaluation of plant height, aerial biomass, yield, and yield-related parameters, a total of five normal and competitive plants were selected.

Several environmental covariates were calculated to evaluate the effect of environmental conditions on determining grain yield under several stressed conditions. To evaluate the effect of soil moisture stress on final grain yield, we calculate the cumulative hydric deficit (DHC, mm) as follows [26]:


DHC = Irrigation + Precipitation – ETP (1)

ETP represents the potential evapotranspiration and can be estimated using different models such as Hargreaves or Penman-Monteith. In our study, ETP (mm/day) was calculated following Hargreaves Method as follows [26]:


$$\text{ETP}\;=\;\text{0}.\text{0023}\;\times \;(\text{T}\_\text{avg}\;+\;\text{17}.\text{8})\;\times \;\sqrt{}\;(\text{T}\_\text{max}\;-\;\text{T}\_\text{min})\;\times \;\text{Ra}$$ (2)

With 17.8 is a constant used in the equation for estimating potential evapotranspiration (ETP) and 0.0023 an empirical adjustment constant introduced by Hargreaves and Samani [26] to align the estimated evapotranspiration with observed data under various climatic conditions. T_avg, T_max, and T_min represent the average, maximum, and minimum temperatures of each environment, respectively, and Ra is the extraterrestrial radiation (MJ/m^2^/day), which depends on the latitude and the day of the year. The Water stress index (WSI) was also calculated to compare the available water and the water requirements of the maize plants. WSI was calculated as follows [26]:


WSI = (Irrigation + Precipitation) / ETP (3)

On the other hand, DAY > 40 and ASI_ENV, representing respectively the total number of days when the temperature exceeded 40 degrees in each environment and the average anthesis silking interval of the different genotypes in each environment, were also calculated to estimate the effect of heat stress.

### Statistical analysis

Analyses of variance were performed for each trait. The sources of variation were environment (E), bloc within the environment (bloc(environment)), genotypes (G), and genotype × environment interaction (G × E). G, E, and G × E were considered fixed effects; while blocks nested within environments (bloc(environment)) was considered random. A combined analysis of variance over environments was firstly performed. Subsequently, individual analysis of variance including the checks were made for each environment for the traits showing significant genotype × environment interaction. The least significant difference (LSD) at P < 0.05 was calculated using the checks to compare the LS means (Least Squares Means) of the landraces. This part of the statistical analysis was made using the SAS Proc Mixed procedure [27–30]. For traits showing a significant G × E interaction, separate analyses of variance were conducted within each environment while for traits without significant interaction, the LSD was derived from the combined analysis across environments.

The GGE biplot method was employed to analyze the interaction and stability of genotypes when a significant G × E was detected. The GGE biplot was constructed using the first two principal components (PC1 and PC2), derived from environment-centered yield data. The advanced multivariate analyses, including “mean vs. stability” biplot and “which won where” biplot analyses, were accomplished using the “metan” package version 1.18.0 [27], with R version 4.1.2 in RStudio [31], providing a clear visualization of the genotype × environment interactions and the stability of the genotypes across environments [32]. The analyses were based on the single portioning value (SVP) of the first two principal components as follows:


Yij = μ + αi + βj + θij + ϵij

Where is observation of genotype i in environment (Yij) is the mixed effect value of grand mean (μ), which is modified by the main effect of genotype (αi), environment main effect (βj), as well as the interaction between the genotype and environment due to ith genotype and jth environment (θij), and random error (εij) [16].

The heatmap is created using the mean values of the parameters measured under the different growth conditions for each genotype. Hierarchical clustering is applied to the rows (genotypes and environments) and columns (parameters). Using the Euclidean distance, a hierarchical clustering was made to identify similarities. The parameter values are normalized by column and represented by colors, ranging from blue (low values) to red (high values). The heatmap and dendrogram construction were performed with the heatmap R library with RStudio [33].

Factorial regression analyses were performed to evaluate the impact of the environmental (E), genotypic (G), and genotype × environment interaction (G × E) factors contributing to variability among maize grain yield under different drought and heat stress conditions. Factorial regression analysis was performed using R software. The models were fitted with the lm() function from the stats package, and significance of main and interaction effects was assessed using the Anova function from the car package [34].

The general form for a factorial regression model with genotypic and environmental covariates is expressed as described as follows [35]:


$$\text{Yij}\;=\;\mu \;+\;[\Sigma \rho \text{k}\cdot \text{Gik}\;+\;\alpha \text{i}+\;[\Sigma \delta \text{h}\cdot \text{Ejh}\;+\;\beta \text{j}]\;+\;[\Sigma \text{Gik}\cdot \theta \text{kh}\cdot \text{Ejh}\;+\;\Sigma \alpha^{\prime }\text{ih}\cdot \text{Ejh}\;+\;\Sigma \beta^{\prime }\text{jk}\cdot \text{Gik}\;+\;\epsilon \text{ij}],$$

Where ρk and δh are the regression coefficients of genotypic (Gik) and environmental (Ejh) covariates, respectively; αi and βj are the residuals of genotype and environmental main effects, respectively; θkh is the regression coefficient of the cross-product of covariates (Gik and Εjh), and α′ih and β′jk are the genotype-specific (i) and environment-specific(j) regression coefficient of environmental covariate Εjh and genotypic covariate Gik, respectively. εij is the residual interaction effect. In our study, the genotype-specific regression coefficient (α′ih) and the environment-specific regression coefficient (β′jk) were not explicitly estimated but were accounted for within the general residual error term (εij). This approach simplifies the model while maintaining a robust estimation of the G × E interaction effects [36].

## Results

The analysis of variance revealed significant differences among genotypes (GEN), and among environments (ENV) for all traits (S3 Table). Significant ENV × GEN interactions were registered for days to tasseling and silking (DT and DS), plant height (PH), thousand-grain weight (1000GW), and grain yield per plant (GYP), (P < 0.001), suggesting that genotype performance varies across environments (Table 3).

**Table 3:** Mean squares from the analyses of variance of plant height (PH), aerial biomass (AB), number of grains per ear (NG), number of ears (NE), thousand grain weigh (1000GW), number of grains per plant (NGP), grain yield per plant (GYP), anthesis silking interval (ASI), days to tasseling (DT), and days to silking (DS) in nine Tunisian landraces under six environments.

Source	DF	PH (cm)	AB (g)	NG	NE	1000GW (g)	NGP	GYP (g/plant)	ASI (days)	DT	DS
ENV	5	75482.32**	3331376.93**	1049202.00**	10.59**	866501.34**	9368657.85**	3901002.35**	109.85**	1274.43**	2022.14**
BLOC (ENV)	24	6577.04	311412.46	71393.13	2.32	60016.97	383053.27	172824.45	10.40	57.26*	98.46*
GEN	13	27167.24**	346543.57*	194962.31**	4.32**	290775.93**	1576315.06**	820934.73**	38.71**	100.14**	197.34**
GEN × ENV	51	22324.96**	534351.02	168953.00	4.82	322683.23**	1850220.80	1041780.49**	31.33	228.98**	309.42**
Error	56	12554.00	714959.00	218252.00	3.66	101020.00	1759373.00	390867.00	25.20	54.33	96.73

ns: no significant, *: significant at P = 0.05, and **: significant at P = 0.01.

The trials were conducted in six distinct environments representing different climatic conditions in Tunisia during the 2022 and 2023 seasons. Two environments at Mornag (C_22 and C_23) correspond to conditions of moderate heat stress, with maximum temperatures above 32°C and high solar radiation (>39 MJ/m^2^/d) (Table 4). Total potential evapotranspiration (ETP) exceeds 1860 mm, while the quantity of water supplied (W) remained lower, which led to a moderate cumulative hydric deficit (DHC: −320 to −417 mm) and water stress indices (WSI) of around 0.8. On the other hand, in the combined moderate heat and drought stress scenarios at Mornag (S_22 and S_23), water inputs were significantly reduced (1080.53 mm and 1111.36 mm), which resulted in a hydric deficit exceeding −780 mm and a WSI of 0.58, indicating a more severe stress (Table 4). The environment (D_H), exposed to high temperatures (T_MED = 26°C), high radiation (33.5 MJ/m^2^/d), and very low rainfall (15 mm), was the most critical, combining severe heat and water stress with a WSI of 0.43 and a DHC close to −950 mm. In contrast, the environment at Sousse (OP) represented the optimum conditions for the system (Table 4). It was characterized by more moderate average temperatures (22.83°C), a lower ETP (1286.72 mm), a high-water input (1616.08 mm), and an absence of extreme days (>40°C), which reflected ideal conditions for maize development (Table 4).

**Table 4:** Agro-climatic description and abiotic stress levels of experimental environments where the Tunisian maize landraces were evaluated.

Location	Year	Environment	T_Min (ºC)	T_Max (ºC)	T_MED (ºC)	RA (MJ/m²/day)	ETP (mm)	W (mm)	DHC (mm)	WSI	ASI_ENV (days)	DAY > 40	Growth Conditions
Mornag	2022	C_22	17.18	32.33	24.75	39.43	1863.84	1543.42	−320.42	0.83	1.2	25	Moderate heat stress
Mornag	2022	S_22	17.18	32.33	24.75	39.43	1863.84	1080.53	−783.31	0.58	3.0	25	Moderate combined heat and drought stress
Mornag	2023	C_23	20.12	34.45	27.29	39.76	1925.02	1507.34	−417.68	0.78	2.0	29	Moderate heat stress
Mornag	2023	S_23	20.12	34.45	27.29	39.76	1925.02	1111.36	−813.66	0.56	3.7	29	Moderate combined heat and drought stress
Gabes	2022	D_H	20.23	31.68	25.95	33.50	1657.71	710.02	−947.69	0.43	4.3	11	Severe combined heat and drought stress
Sousse	2022	OP	19.08	26.57	22.83	32.89	1286.72	1616.08	329.35	1.26	1.0	–	optimal conditions

The comparison of means revealed a wide diversity among landraces for growth and productivity (Table 5). Under the stressed environments S_22, S_23, and D_H, the landraces BK, KAR, and MT2 consistently demonstrated strong adaptation and high performance across several traits. In particular, BK and MT2 maintained early growth cycles with good anthesis–silking synchrony, flowering at 42 days for tasseling and silking in BK and at 45 and 46 days for DT and DS in MT2 under OP, compared with 44 and 45 days for the checks. Even under stress, they remained earlier than the checks: in S_22, BK and MT2 flowered about one to two days earlier, while under the most stressful environment (D_H) they were still one day earlier for DT and three to four days earlier for DS. Regarding plant height, the tallest genotypes under OP were BK (217 cm), KAR (215 cm), and MT2 (205 cm). Although reduced by 355–44% under D_H, they still remained taller than the checks, which averaged only 132 cm. A similar trend was observed for grain yield per plant. Despite severe reductions between OP and D_H (91% in BK, 92% in KAR, and 89% in MT2), these landraces consistently ranked among the top-performing genotypes under stress. In contrast, GAF, while highly productive under OP and well-watered conditions, exhibited the sharpest yield decline, with a 94% reduction under D_H. Thousand-grain weight (1000GW) followed the same pattern. Under D_H, BK, KAR, and MT2 maintained relatively higher values (276 g, 247 g, and 271 g, respectively) compared with the check average (241 g), whereas GAF dropped to 239 g.

**Table 5:** Comparisons of means for agronomic traits with significant genotype environment interactions as plant height (PH), thousand grain weigh (1000GW), grain yield per plant (GYP), days to tasseling (DT), and days to silking (DS) recorded from the nine Tunisian maize landraces evaluated under six different conditions (C_22, C_23, D_H, OP, S_22, and S_23) in Tunisia.

GEN	PH (cm)						1000GW (g)						GYP (g)					
ENV	C_22	C_23	D_H	OP	S_22	S_23	C_22	C_23	D_H	OP	S_22	S_23	C_22	C_23	D_H	OP	S_22	S_23
BIZ1	172.0	192.0	82.7	191.3	126.0	142.0	546,7	450,3	253,7	570.33	**339.33**	328.00	563.20	420.42	42.05	692.63	115.80	146.00
BIZ2	188.3		84.0	185.0	154.7		414,7		**249,7**	457.00	**348.00**		173.11		48.61	525.74	121.60	
BK	**230.0**	**214.0**	**142.0**	**217.3**	**191.7**	**177.0**	**595**	**482**	**275,7**	**632.67**	**374.67**	338.70	**737.40**	**527.30**	**93.38**	**1060.00**	**243.00**	**304.70**
GAB1	191.3	178.0	**124.5**	186.0	**164.7**	136.0	**637,3**	440	233,3	**618.33**	**340.67**	305.00	582.41	378.27	39.67	**822.70**	174.60	100.00
GAB2	171.0		101.5	162.0	133.3		328,7		205	394.00	237.33		146.35		71.12	291.33	53.58	
GAF	179.7	191.7	77.7	192.6	155.7	148.3	**580**	433,7	239,3	**598.67**	**349.33**	300.30	**785.80**	**422.00**	59.02	**920.60**	145.00	186.60
KAR	**238.7**	**206.0**	**132.3**	**215.0**	**194.7**	**163.7**	**672,7**	**498**	**247,3**	**642.00**	**364.67**	**449.00**	**689.40**	**493.70**	**76.71**	**958.40**	**256.00**	**351.70**
MT1	187.7	184.3	**122.7**	189.6	**163.0**	139.0	548,3	426	211,3	**638.00**	285.33	311.30	626.18	383.81	53.25	714.75	173.70	148.40
MT2	**220.3**	**194.7**	**115.4**	**205.3**	**180.3**	**158.0**	**578**	450,6	**271,3**	**619.33**	**320.00**	284.00	**750.50**	**431.80**	**98.67**	**870.70**	**253.00**	182.00
**Checks**																		
AGRISTER	202.3	206.7	134.8	202.8	157.0	144.3	385.70	428.40	277.00	450.73	362.93	392.70	407.76	444.00	55.90	535.83	162	265,4
FANDARI	226.1	230.9	126.1	217.7	160.8	146.1	366.80	473.80	221.70	411.93	268.27	346.40	326.77	575.84	54.44	538.58	139,6	232
SANCIA	206.2		135.3	154.5	176.8		357.70		225.20	386.87	287.67		309.77		43.24	541.13	143,5	
B73 × Mo17		225.1				175.9		431.73				370.10		468.67				251,6
BS17		197.9				132.9		453.27				400.20		542.73				236,1
Mean Check	211.6	215.1	132.1	191.7	164.9	151.6	370.10	446.80	241.30	416.51	306.29	372.30	348.10	507.81	51.20	538.51	148.30	239.90
LSD	33.4	40.2	53.6	56.6	37.6	24.1	194.90	70.90	49.33	164.82	112.68	92.68	130.38	206.82	39.67	489.45	75.32	125.50
**GEN**	**DT**						**DS**											
ENV	C_22	C_23	D_H	OP	S_22	S_23	C_22	C_23	D_H	OP	S_22	S_23						
BIZ1	**45.0**	**50.0**	54.0	47.0	55.0	54.0	**46.0**	52.0	59.0	49.0	61.0	61.0						
BIZ2	48.0		55.0	45.0	51.0		51.0		61.0	47.0	55.0							
BK	**45.0**	**49.0**	**52.0**	**42.0**	**46.0**	**52.0**	**45.0**	**50.0**	**55.0**	**42.0**	**49.0**	**54.0**						
GAB1	**45.0**	54.0	54.0	45.0	**47.0**	57.0	**46.0**	57.0	59.0	46.0	**50.0**	61.0						
GAB2	46.0		55.0	**42.0**	55.0		48.0		60.0	45.0	61.0							
GAF	**45.0**	51.0	53.0	**42.0**	49.0	55.0	**46.0**	53.0	57.0	**42.0**	52.0	58.0						
KAR	**45.0**	**50.0**	54.0	45.0	51.0	**54.0**	**46.0**	**51.0**	59.0	45.0	54.0	**56.0**						
MT1	**45.0**	54.0	**52.0**	**42.0**	49.0	57.0	**46.0**	56.0	**56.0**	**42.0**	52.0	63.0						
MT2	**45.0**	54.0	**51.0**	45.0	**47.0**	56.0	**45.0**	57.0	**54.0**	46.0	**49.0**	60.0						
**Checks**																		
AGRISTER	45.0	51.0	53.0	44.0	47.8	56.0	46.6	52.2	56.8	45.0	50.8	59.2						
FANDARI	45.0	53.0	52.6	45.0	47.8	60.0	47.4	54.4	56.6	46.0	51.0	62.6						
SANCIA	45.0		54.4	43.0	48.0		47.2		59.8	45.0	51.4							
B73 × Mo17		52.6				57.4		53.6				60.2						
BS17		51.0				58.0		52.4				60.6						
Mean Check	45.0	51.9	53.3	44.0	47.9	58.5	47.1	53.2	57.7	45.0	51.1	61.1						
LSD	0.0	3.6	2.3	4.4	2.7	3.3	1.8	4.3	3.8	6.1	3.9	3.9						

The values in bolt signify that the landraces were not significantly different from the highest performant landrace, based on mean comparisons using least significant difference (LSD) test at a P-value < 0.05.

Overall, the checks showed an intermediate level of performance across environments. They tended to flower later and produce taller plants under optimal conditions, but were less resilient under stress compared with the best-performing landraces. BK, KAR, and MT2 flowered earlier than the checks. Under D_H, BK and KAR consistently surpassed them in grain yield (Table 5).

**Fig 3:**
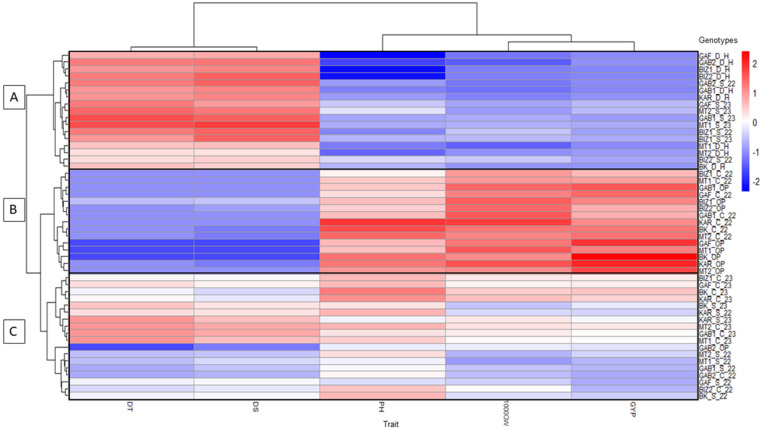
Heatmap representing hierarchical clustering of five measured traits for nine Tunisian maize landraces based on the significant rate change values in response to several environmental conditions (C_22, C_23, S_22, S_23, OP, and D_H), A, B, and C are the clusters based on Euclidean distance measurement for the landraces. The color bar depicts the gradient of rate change values with Red tones representing high performance and blue tones indicating the low performance in response to different conditions for GYP: grain yield per plant (g), PH: plant height (cm), 1000GW: thousand-grain weight (g), DS: days to silking, and DT: days to tasseling.

The heatmap (Fig 3) classified the genotypes into three main clusters according to their responses to different stress levels. Cluster A grouped environments with severe drought and heat stress (D_H) or moderate drought combined with heat waves (S_22 and S_23), where most genotypes showed strong yield reductions, except for a few that maintained flowering synchronization or moderate yield stability. Cluster B included favorable environments (OP and C_22), characterized by intense red tones for plant height and yield traits, contrasting with cooler tones for flowering parameters, highlighting better overall growth. Cluster C comprised genotypes under intermediate stress (C_23 and S_23), with more variable performance but still allowing some genotypes to maintain relatively high yields compared to their optimal performance.

The heatmap confirmed the mean comparison patterns (Table 5). The landraces MT1, GAB1 and BIZ1 showed limited adaptability, while BK, KAR, and MT2 combined early flowering synchronization with yield stability, particularly under stress. These trends were corroborated by the clustering analysis (S1 Fig).

Considering the overall performance of the genotypes across all environments for the traits with no significant G × E, KAR and BK were the top-performing genotypes (Table 6). KAR recorded the highest aerial biomass (592.75 g), number of grains per plant (862.79), coupled with a low anthesis-silking interval (1.99 days). Similarly, BK achieved high aerial biomass of 587.41 g, the highest NGP of 1059.97, and the shortest ASI with 1.21 days, marking it as a superior genotype for yield potential (Table 6). GAF, MT1, and MT2 fall within an intermediate category of populations, showing balanced performance without extreme variability. GAF has a commendable NGP of 867.72 and a moderate average anthesis silking interval of 2.10 days, while BIZ1 and BIZ2 exhibited stability across traits, maintaining moderate-to-high values for AB and NGP with medium ASI between 2.20 and 2.39, days respectively (Table 6).

**Table 6:** Comparisons of means of the average six environments for agronomic traits with no significant genotype environment interactions as aerial biomass (AB), number of grains per ear (NG), number of ears (NE), number of grains per plant (NGP), and anthesis silking interval (ASI) recorded from the nine Tunisian maize landraces.

GEN	AB (g)	NG	NE	NGP	ASI (days)
BIZ1	422.82	**421.68**	1.37	639.01	3.34
BIZ2	354.92	335.75	1.42	471.67	3.75
BK	**587.41**	**451.32**	**2.27**	**1059.97**	**1.21**
GAB1	**491.04**	**389.95**	1.54	660.41	2.83
GAB2	309.33	312.42	1.42	435.83	4.25
GAF	430.92	**450.78**	1.83	867.72	2.10
KAR	**592.75**	**408.83**	**2.03**	**872.48**	**1.99**
MT1	464.60	**407.52**	1.92	821.94	2.39
MT2	**479.28**	**420.67**	**2.10**	**899.11**	**2.20**
Checks					
AGRISTER	501.21	409.56	1.77	764.92	2.40
FANDARI	506.19	463.31	1.67	816.67	2.47
SANCIA	509.23	373.00	1.83	741.12	3.10
B73 × Mo17	550.70	485.43	1.80	882.63	1.90
BS17	521.02	490.33	1.78	898.42	2.00
Mean check	517.67	444.33	1.77	820.75	2.37
LSD	120.48	66.57	0.27	189.00	0.72

The values in bolt signify that the landraces were not significantly different from the highest performant landrace, based on mean comparisons using least significant difference (LSD) test at a P-value < 0.05.

**Fig 4:**
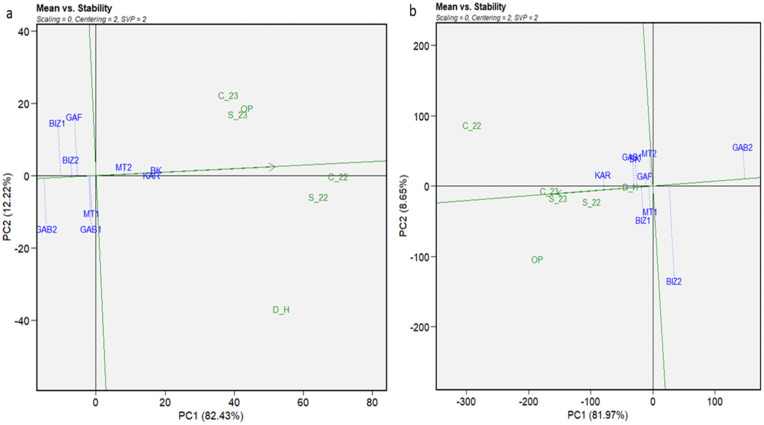
GGE biplot of mean performance vs. stability for plant height (cm) (a) and thousand-grain weight (b) for nine Tunisian maize landraces under six environmental conditions (C_22, C_23, S_22, S_23, OP, and D_H).

**Fig 5:**
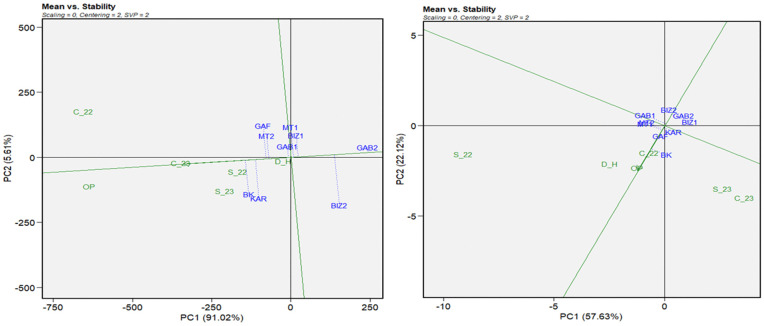
GGE biplot of mean performance vs. stability for grain yield per plant (g) (a) and days to tasseling (b) for nine Tunisian maize landraces under six environmental conditions (C_22, C_23, S_22, S_23, OP, and D_H).

**Fig 6:**
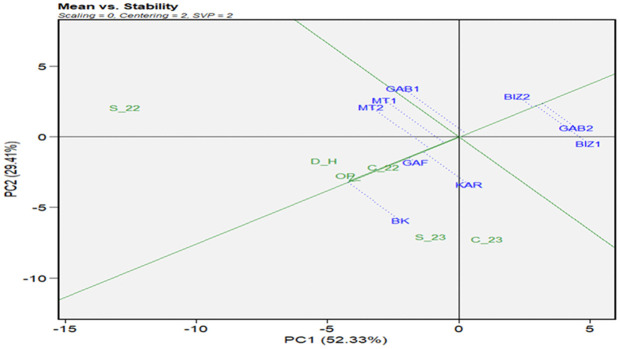
GGE biplot of mean performance vs. stability for days to silking for nine Tunisian maize landraces under six environmental conditions (C_22, C_23, S_22, S_23, OP, and D_H).

The GGE biplot of means vs. stability (Fig 4) described a graphical representation of the mean performance and stability of tested genotypes across environments with genotypic matrix (SVP = 2) and environment-centered (centering = 2). The first two principal components (PC1 and PC2) explained 95% of the total variation for plant height. The genotypes BK and KAR, followed by MT2, were the most performing genotypes for PH, and showed higher stability across the different environments (Fig 4-a). For 1000GW (Fig 4-b), where the first principal components explained 91% of the total variation, KAR was the most performant genotype, followed by BK and GAB1. For stability, the most stable genotype for 1000GW was GAF, followed by KAR and MT1. The means vs. stability of the grain yield (Fig 5-a) showed that the two first principal components explained 97% of the total variation. BK and KAR were the most performant genotypes, followed by GAF and MT2, while GAB2 was the less performant and the most stable genotype. The GGE means vs stability of the number of days to tasseling (Fig 5-b), the total variation represented by the two first principal components was 80% of the total variation. GAF and BIZ2 were the most stable genotypes. BK, followed by GAF, registered the highest performance as the earliest genotype, while BIZ2, which represented significant stability, was the less performant and latest genotype, followed by GAB2. The results of the number of days to silking (Fig 6) highlighted GAF again as the most stable for the number of days to silking the principal components, explaining 82% of the total variation, followed by BIZ2 and GAB2. BK, KAR, and MT2 did not register the highest stability; but they exhibited the earliest silking performance. In contrast, GAB2 and BIZ2 with high stability were the latest-flowering genotypes.

**Fig 7:**
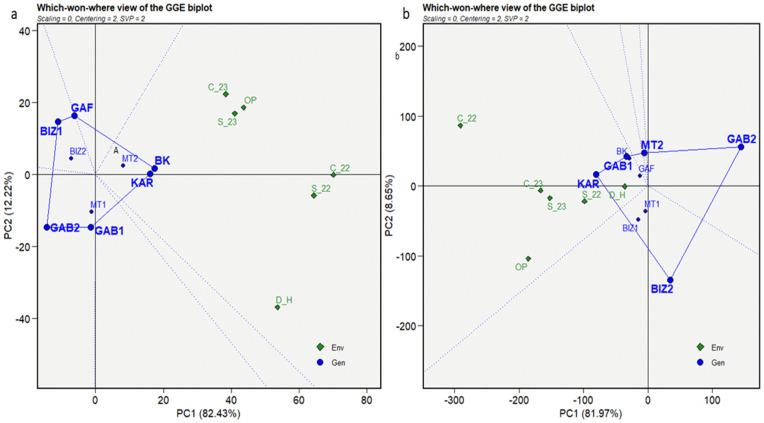
Which-won-where view of the GGE biplot for plant height (cm) (a) and thousand-grain weight (g) (b) for nine Tunisian maize landraces under six environmental conditions (C_22, C_23, S_22, S_23, OP, and D_H).

The insights obtained from the mean vs. stability biplots prepare a foundation for further analysis of the superior performance of the genotypes under distinct environments, leading us to the “Which Won Where” biplot analysis. This analysis aimed to identify the high-performing, adapted, and stable genotypes under different stress conditions. A polygon connecting the outermost genotypes formed the convex of the plant height (Fig 7-a). Equality lines drawn from the origin to each side of the biplot divided the biplot into sectors with one genotype at each vertex, representing the best or the poorest performances recorded in one or more environments. Six vertexes were included in the convex of plant height: BK, KAR, GAB1, GAB2, BIZ1, and GAF (Fig 7-a). BK and KAR registered the shortest connector lines, as similar performant genotypes in all environments. In addition, all the environments were placed on the same side of the perpendicular line on the BK sector. The results showed that BK, followed by KAR, were the best and most adapted genotype under overall conditions.

The genotypes KAR, GAB1, MT2, GAB2, and BIZ2 represented the most responsive cultivars and formed the polygon of the thousand-grain weight (Fig 7-b). The short connector line between GAB1 and MT2 reflected their similar performance across all environments. The biplot showed that the environments were positioned on the KAR sector on the same side of the perpendicular line. Thus, KAR became the most performant genotype for 1000GW in all environments, followed by GAB1 and MT2. The genotypes inside the polygon, such as BIZ1, MT1, and GAF, exhibited lower performance than those on the vertices but more stability. GAF was the most stable, as the closest to the origin of the biplot. After reversing 1000GW signs, the resulting biplot showed that all the environments were classified in the GAB2 sector.

**Fig 8:**
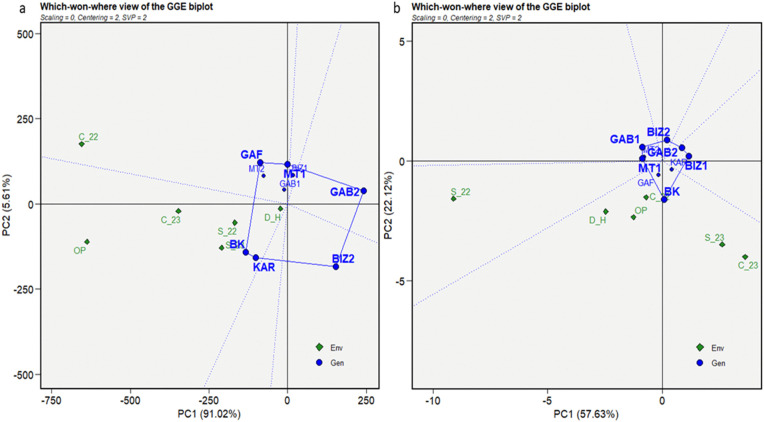
Which-won-where view of the GGE biplot for grain yield per plant (g) (a), days to tasseling (b) for nine Tunisian maize landraces under six environmental conditions (C_22, C_23, S_22, S_23, OP, and D_H).

The “which won where view” of grain yield (GYP) (Fig 8-a) showed a convex composed of, BK, GAF, MT1, GAB2, BIZ2, and KAR representing the extremely best and poorest performer genotypes. The close distance between BK and KAR, closely followed by GAF and MT1, indicated similar performance patterns of these genotypes through all the environments. Most of the environments were figured on the same side of the biplot perpendicular line in the BK sector, except for C_22, which figured in the GAF sector.

**Fig 9:**
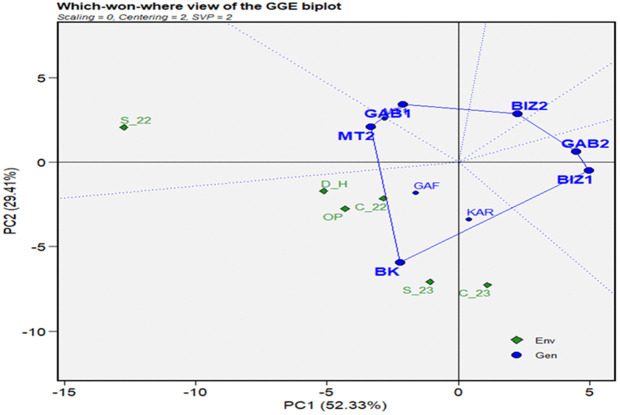
Which-won-where view of the GGE biplot for days to silking for nine Tunisian maize landraces under six environmental conditions (C_22, C_23, S_22, S_23, OP, and D_H).

To further evaluate flowering synchronization in the tested genotypes, the earliest genotypes regarding tasseling (DT) and silking (DS) were identified as those that reached these stages, respectively, before 60 and 65 days. The polygon for DT was formed by the vertex genotypes BK, BIZ1, GAB2, BIZ2, GAB1, and MT1 (Fig 8-b). MT1 was the earliest genotype in the S_22 environment; while the other environments fell into the BK sector. GAB1 and MT1 registered similar performance, as depicted by the short length of their connector vector. The biplot of reversed-sign indicated that BIZ1 and GAB1 were the latest genotypes under the most stressed conditions, while GAB2 was the latest genotype under control environments. The genotypes BK, BIZ1, GAB2, BIZ2, GAB1, and MT2 formed the convex for the number of days to silking, corresponding to the most distant genotypes from the origin (Fig 9). The earliest genotype for the stressed condition of S_22 was MT2, while the other environments figured on the BK sector. The reversed-sign analysis showed that BIZ1, BIZ2, and GAB1 were the latest genotypes for silking under stressed environments, while GAB2 was the latest under control and optimum conditions.

**Fig 10:**
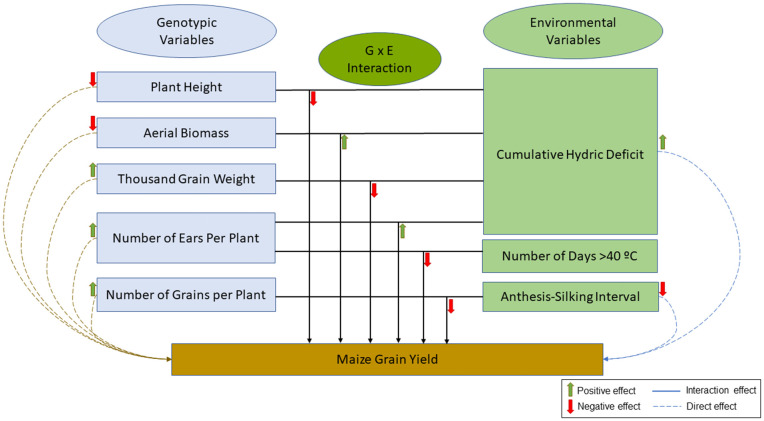
Factorial regression of genotype × environment effects on maize grain yield: Direct and interactive contributions for nine Tunisian maize landraces evaluated under six environmental conditions (C_22, C_23, S_22, S_23, OP, and D_H).

Factorial regression was conducted to provide a biological interpretation of the main effects of genotype (G), environment (E), and their interaction (G × E) on grain yield (Table 7, S2 Fig). The results showed that the significant genotypic covariates were plant height, aerial biomass, number of ears, number of grains per plant, and thousand-grain weight. The genotypic covariates represented 60% of the total genotypic variation. Whereas cumulative hydric deficit (DHC), anthesis silking interval were the most significant environmental covariates representing a total of 48% of the total environmental variation (Table 7). The highest representativeness genotype covariates were plant height followed by number of ears, thousand-grain weight and aerial biomass respectively with 26%, 13%, 9% and 8% of the total variability. The regression coefficients of PH and AB on Grain Yield were negative (αPH= −0.797, αAB=−0.027, respectively), while for NE, NGP, and 1000KW there were positive effects (αNE = 13.361, αNGP = 0.561, and α1000kw=1.082, respectively). The regression coefficients of the environmental covariates were positive for DHC (βDHC = 0.968) and negative for ASI_ENV, as the average ASI of the different genotypes in each environment (βASI_ENV= −65.629). The results exhibited also four significant interactions between several genotypic covariates and the environmental covariate DHC such as, PH × DHC, (θ= −0.0116), AB × DHC (θ = 0.00035), NE × DHC (θ = 0.191), and 1000GW × DHC (θ= −0.013). Significant interactions were also found between NE × DAY > 40 (θ= −0.556) and NGP × ASI_ENV (θ= −0.049). All the significant interacted covariates explained approximately 35% of the sum of squares for G × E (Table 7, Fig 10).

**Table 7:** Analysis of variance of the factorial regression model for grain yield including seven genotypic as the number of days to pollen shedding (DT), number of days to silking (DS), anthesis-silking interval (ASI), plant height (PH), aerial biomass (AB), number of grains per ear (NG), number of grains per plant (NGP), thousand-grain weight (1000GW) and number of ears per plant (NE), With four environmental covariates as the cumulative hydric deficit (DHC), the Water stress index (WSI), total number of days when the temperature exceeded 40 degrees in each environment (DAY > 40),and the average anthesis silking interval of the different genotypes in each environment ASI_ENV. Trials were conducted with nine maize landraces under six different environments.

Source of variation		DF	Sum of squares^†^	Mean of Squares^ŧ^	Estimate^‡^	% variability
GEN		13	13.034	1.003***		
	PH	1	3.395	3.395***	α = −0.797	26.04
	AB	1	1.072	1.072**	α = −0.027	8.22
	NE	1	1.598	1.598***	α = 13.361	12.26
	NGP	1	0.513	0.513*	α = 0.561	3.93
	1000 GW	1	1.204	1.204**	α = 1.082	9.23
	ASI_GEN	1	0.010	0.010		0.07
	DS	1	0.035	0.035		0.26
	Residual GEN	6	5.206	0.868***		39.94
ENV		5	106.245	21.249***		
	DHC	1	48.081	48.081***	β = 0.968	45.25
	ASI_ENV	1	2.845	2.845***	β = −65.629	2.67
	DAY > 40	1	0.092	0.092		0.08
	WSI	1	0.062	0.062		0.05
	Residual ENV	1	55.165	55.165***		51.92
GEN × ENV		51	15.477	0.303***		
	PH × DHC	1	0.836	0.836**	θ = −0.0116	5.40
	PH × ASI_ENV	1	0.253	0.253		1.63
	PH × DAY > 40	1	0.037	0.037		0.23
	PH × WSI	1	0.304	0.347		1.96
	AB × DHC	1	0.642	0.632**	θ = 0.00035	4.14
	AB × ASI_ENV	1	0.209	0.209		1.35
	AB × DAY > 40	1	0.189	0.074		1.22
	AB × WSI	1	0.022	0.001		0.14
	NE × DHC	1	1.100	1.1***	θ = −0.191	7.10
	NE × ASI_ENV	1	0.161	0.161		1.04
	NE × DAY > 40	1	0.570	0.563**	θ = −0.556	3.68
	NE × WSI	1	0.002	0.086		0.01
	NGP × DHC	1	0.248	0.296		1.60
	NGP × ASI_ENV	1	0.743	0.732**	θ = −0.049	4.80
	NGP × DAY > 40	1	0.002	0.000		0.01
	NGP × WSI	1	0.019	0.018		0.12
	1000GW × DHC	1	1.406	1.354***	θ = −0.013	9.08
	1000GW × ASI_ENV	1	0.010	0.014		0.06
	1000GW × DAY > 40	1	0.054	0.055		0.34
	1000GW × WSI	1	0.017	0.000		0.10
	ASI_GEN × DHC	1	0.052	0.043		0.33
	ASI_GEN × ASI_ENV	1	0.032	0.031		0.20
	ASI_GEN × DAY > 40	1	0.002	0.004		0.01
	ASI_GEN × WSI	1	0.046	0.047		0.29
	DS × DHC	1	0.035	0.019		0.22
	DS × ASI_ENV	1	0.110	0.110		0.71
	DS × DAY > 40	1	0.012	0.035		0.07
	DS × WSI	1	0.045	0.069		0.29
	Residual G × E	23	8.319	0.362***		53.75
ERROR		56	7.161	0.128		

R^2^=46.6%

* Significant at 0.05 probability level.

** Significant at 0.01 probability level.

*** Significant at 0.001 probability level.

^†^Sum of squares for the main and the interaction effects were decomposed; sum of squares for each partition of main and interaction effects were expressed as the percentage of the sum of squares of the corresponding main or interaction effect.

^ŧ^Mean of squares for the main and the interaction effects were decomposed; sum of squares for each partition of main and interaction effects were expressed as the percentage of the sum of squares of the corresponding main or interaction effect.

^‡^α and β are the regression coefficients for genotypic and environmental covariates, respectively. θ is the regression coefficient for genotypic and environmental interaction covariates.

## Discussion

All environments, except Sousse (OP), experienced stressful conditions during these evaluations. Even under the well-watered conditions of C_22 and C_23, the heat waves that occurred for several days classified them as stressed environments, which could partially explain the results of the factorial regression. The significant differences among environments, and genotypes confirm the environmental and genetic diversity involved in this study [18]. Additionally, the significant genotype × environment interaction of PH, 1000GW, GYP, DT, and DS illustrated on the GGE biplot analysis indicated the presence of crossover interactions for those traits [36]. These interactions resulted in variable responses under different environmental conditions, confirming the effect of genetic variability among landraces [37].

Differences among genotypes were significant for number of days to tasseling and silking across environments, highlighting its critical role in selecting tolerant maize genotypes under different stress conditions. Several studies reported similar findings, supporting this perspective [12,38]. The largest ASI was recorded under the combined drought and heat stress conditions. Conversely, the shortest ASI was recorded under optimal moisture conditions. These results align with previous studies showing that heightened stress levels are reflected in increased ASI and flowering-synchronization problems, whereas a shorter ASI indicates favorable growing conditions [39,40]. Additionally, the negative relation of ASI with GYP and yield components (1000GW, NE, NGP). highlights that delayed flowering with extended ASI cause reproductive delays with an inefficient resource allocation, leading to lower yields [41].

The biological importance of a short ASI lies in maintaining synchrony between pollen shedding and silk emergence, which is essential for efficient pollination. In contrast, an extended ASI disrupts fertilization, increases ovary abortion, and reduces assimilate allocation to kernels, leading to lower yields [38,41]. Different levels of grain yield reduction were observed under varying stress scenarios, and the high decreases were registered under the combined drought and heat stress conditions. This observation aligns with findings from previous studies documenting the variability of grain yield under several stress levels [37,42].

BK stood out with the shortest ASI across all environments, contributing to its earliness, higher number of ears per plant, and high grain yield. Similarly, MT2 and KAR also maintained short ASI values, which translated into superior yield performance. Previous reports concluded that flowering synchronization and earliness could significantly affect yield stability and adaptability [43], particularly in Mediterranean conditions where flowering often coincides with episodes of drought and heat stress.

For several traits, these three landraces emerged as the top-performing genotypes and even outperformed some checks, suggesting they could be promising sources of favorable alleles for breeding programs focusing on sustainable maize production in arid and semiarid environments. Their consistent high performance across environments and traits highlights strong adaptability and resilience. Previous studies reported that the genetic background of BK, MT2, and KAR shared similarities with reference populations such as Reid Yellow Dent and Lancaster Sure Crop, both recognized for their adaptability to severe environmental conditions [23,44].The factorial regression analysis showed that grain yield per plant (GYP) is determined by agronomic traits, environmental variables, and their interactions. Among the main effects, plant height (PH) and aerial biomass (AB) showed negative associations with yield (Fig 10). This result suggests their main direct contribution was already explained by other yield-related traits such as 1000GW and NGP. Once the other traits are considered, the remaining effect of PH and AB reflects mainly the cost of maintaining excessive vegetative growth, which limits resource availability for reproductive development and reduces grain yield (Fig 10). In contrast, the yield components such as the number of ears (NE), the number of grains per plant (NGP), and thousand-grain weight (1000GW) showed significant positive effects, confirming their central role in determining yield [45].

Regarding environmental factors, cumulative hydric deficit (DHC) exerted a strong positive effect, highlighting the direct dependence of yield on water supply. Otherwise, an increase in the anthesis–silking Interval in the environment (ASI_ENV), a key indicator of reproductive stress, was strongly associated with yield reduction, reflecting the negative impact of stress on fertilization and grain set [38].

The analysis of interactions further refines this understanding and highlights the adaptive plasticity of genotypes under abiotic constraints. The PH × DHC interaction does not reflect changes in plant height after stress, since stem elongation ended before flowering and the water stress was imposed 14 days earlier. Instead, it shows that the effect of an already established plant height on yield depends on water availability. Under water stress, taller genotypes maintain higher grain yield, indicating that early vigor and greater plant height before flowering provide an adaptive advantage [46].

The AB × DHC and NE × DHC interactions suggest that reducing biomass and number of ears under stress represents a resource-saving strategy, limiting vegetative and reproductive load and reallocating assimilates to the remaining ears [47,48]. The 1000GW × DHC interaction indicates that, under stress conditions, yield can be partly compensated by an increase in grain weight, reflecting enhanced grain filling [43]. In addition, NGP × ASI_ENV and NE × DAY > 40 confirmed that, under combined water and heat stress, plants tend to reduce the number of grains per ear and the number of ears per plant [49,50]. Although this reduces reproductive potential, it favors the development of the remaining grains. This adaptive strategy of reducing reproductive load thus secures yield under extreme stress conditions.

Overall, these results indicate that stress tolerance relies on a differentiated resource allocation mechanism. Under limiting conditions, plants reduce its aerial biomass and prioritize reproductive functions over vegetative growth. They secure grain quality rather than grain number, increasing thousand-grain weight (1000GW) while reducing the number of ears per plant (NE) and grains per plant (NGP). This adaptive strategy reflects a targeted use of an available resources to produce fewer but better-developed grains, thereby ensuring the maintenance of a minimum productivity under stress.

From a breeding perspective, several traits emerged as key contributors to stress resilience and productivity. Traits such as ASI, 1000GW, NE, and NGP showed strong interactions with environmental stress factors and were consistently associated with yield stability. Genotypes like BK, KAR, and MT2 combined short ASI, high NE and NGP, and consistent grain weight, indicating their potential as parental lines in breeding programs. Future breeding efforts should prioritize these traits and explore potential crosses between elite landraces to accumulate favorable alleles conferring drought and heat tolerance.

## Conclusion

The Tunisian maize landraces BK, KAR, and MT2 demonstrated strong adaptation, maintaining shorter anthesis–silking intervals, high grain number per plant, and moderate reductions in plant height and grain yield under combined drought and heat stress. Significant genotype × environment interactions highlighted that extended ASI, reduced water availability, and frequent high-temperature days negatively impacted grain set and yield. These stresses activated adaptive resource allocation strategies to promote grain yield, whereby plants limited vegetative growth and reduced ear and grains number, while prioritizing the filling of kernels to ensure grain quality under adverse conditions. From a breeding perspective, short ASI, high NGP, moderate plant height, and stable grain weight emerged as key traits for improving yield resilience under combined drought and heat stress. The landraces BK, KAR, and MT2 represent promising donor lines for pre-breeding programs. Future research should integrate these landraces into selection pipelines through marker-assisted or genomic prediction tools while incorporating root system architecture, physiological indicators, and molecular markers to better characterize adaptive mechanisms and accelerate selection. Beyond Tunisia, these findings provide valuable insights for Mediterranean drylands and other drought- and heat-prone regions worldwide, offering practical genetic resources to develop resilient cultivars that secure yield stability and enhance food security under climate stress.

## Supporting information

S1 Fig Hierarchical Clustering Dendrogram of nine Tunisian landraces under six different environments using Ward’s Method (ward. D2). (TIF)

S2 Fig Residual plots of the analysis of variance of the factorial regression model for grain yield. (TIF)

S1 Table Comparisons of means for agronomic traits with no significant genotype environment interactions as anthesis silking interval (ASI), aerial biomass (AB), number of ears per plant (NE), number of grains per ear (NG), and number of grains per plant (NGP) recorded from the nine Tunisian maize landraces evaluated under six different conditions (C_22, C_23, D_H, OP, S_22, and S_23). (XLSX)

S2 Table Principal Component Scores (PC1 and PC2) of nine Tunisian Landraces Grouped by Genotypic and Environmental Conditions. (XLSX)

## Abbreviations

DT: Days to Tasseling
DS: Days to Silking
ASI: Anthesis Silking interval
PH: Plant height
AB: Aerial biomass
NG: Number of grains per ear
NGP: Number of grains per plant
1000GW: Thousand-grain weight
NE: Ears per plant
GYP: grain yield per plant
DHC: Cumulative hydric deficit
WSI: Water Stress Index
ETP: Potential evapotranspiration
Ra: Extraterrestrial radiation
T_ Avg: Average temperatures during the growth cycle
T_Max: Maximum temperatures during the growth cycle
T_Min: Minimum temperatures during the growth cycle
DAY > 40: Total number of days when the temperature exceeded 40 degrees
ASI_ENV: Average anthesis-silking interval of all the landraces evaluated in one environment
G: Genotype
E: Environment
G x E: Genotype × Environment interaction

## References

[1] Khan NA, Yu P, Ali M, Cone JW, Hendriks WH. Nutritive value of maize silage in relation to dairy cow performance and milk quality. J Sci Food Agric. 2015;95(2):238–52. doi: 10.1002/jsfa.6703 24752455

[2] Singh A, Pandey H, Pandey S, Lal D, Chauhan D, Aparna, et al. Drought stress in maize: stress perception to molecular response and strategies for its improvement. Funct Integr Genomics. 2023;23(4). doi: 10.1007/s10142-023-01226-6 37697159

[3] Hussain HA, Men S, Hussain S, Chen Y, Ali S, Zhang S, et al. Interactive effects of drought and heat stresses on morpho-physiological attributes, yield, nutrient uptake and oxidative status in maize hybrids. Sci Rep. 2019;9(1):3890. doi: 10.1038/s41598-019-40362-7 30846745 PMC6405865

[4] Dong X, Guan L, Zhang P, Liu X, Li S, Fu Z, et al. Responses of maize with different growth periods to heat stress around flowering and early grain filling. Agricult Forest Meteorol. 2021;303:108378. doi: 10.1016/j.agrformet.2021.108378

[5] Rida S, Maafi O, López-Malvar A, Revilla P, Riache M, Djemel A. Genetics of germination and seedling traits under drought stress in a MAGIC population of maize. Plants (Basel). 2021;10(9):1786. doi: 10.3390/plants10091786 34579319 PMC8468063

[6] Serna L. Maize stomatal responses against the climate change. Front Plant Sci. 2022;13:952146. doi: 10.3389/fpls.2022.952146 36204083 PMC9531676

[7] Huang C, Qin A, Gao Y, Ma S, Liu Z, Zhao B, et al. Effects of water deficit at different stages on growth and ear quality of waxy maize. Front Plant Sci. 2023;14:1069551. doi: 10.3389/fpls.2023.1069551 36818831 PMC9930991

[8] Yi B, Zhou Y, Gao M, Zhang Z, Han Y, Yang G, et al. Effect of Drought Stress During Flowering Stage on Starch Accumulation and Starch Synthesis Enzymes in Sorghum Grains. Journal of Integrative Agriculture. 2014;13(11):2399–406. doi: 10.1016/s2095-3119(13)60694-2

[9] Deribe H. Review on Effects of Drought Stress on Maize Growth, Yield and Its Management Strategies. Communications in Soil Science and Plant Analysis. 2024;56(1):123–43. doi: 10.1080/00103624.2024.2404663

[10] Széles A, Horváth É, Simon K, Zagyi P, Huzsvai L. Maize Production under Drought Stress: Nutrient Supply, Yield Prediction. Plants (Basel). 2023;12(18):3301. doi: 10.3390/plants12183301 37765465 PMC10535841

[11] Kim K-H, Lee B-M. Effects of Climate Change and Drought Tolerance on Maize Growth. Plants (Basel). 2023;12(20):3548. doi: 10.3390/plants12203548 37896012 PMC10610049

[12] Kamkar B, Feyzbakhsh MT, Mokhtarpour H, Barbir J, Grahić J, Tabor S, et al. Effect of heat stress during anthesis on the Summer Maize grain formation: Using integrated modelling and multi-criteria GIS-based method. Ecological Modelling. 2023;481:110318. doi: 10.1016/j.ecolmodel.2023.110318

[13] Notununu I, Moleleki L, Roopnarain A, Adeleke R. Effects of plant growth-promoting rhizobacteria on the molecular responses of maize under drought and heat stresses: A review. Pedosphere. 2022;32(1):90–106. doi: 10.1016/s1002-0160(21)60051-6

[14] Kulkarni AP, Tripathi MP, Gautam D, Koirala KB, Kandel M, Regmi D, et al. Impact of adoption of heat-stress tolerant maize hybrid on yield and profitability: Evidence from Terai region of Nepal. Front Sustain Food Syst. 2023;7. doi: 10.3389/fsufs.2023.1101717

[15] Katsenios N, Sparangis P, Leonidakis D, Katsaros G, Kakabouki I, Vlachakis D, et al. Effect of genotype × environment interaction on yield of maize hybrids in greece using AMMI analysis. Agronomy. 2021;11(3):479. doi: 10.3390/agronomy11030479

[16] Yan W, Kang MS, Ma B, Woods S, Cornelius PL. GGE Biplot vs. AMMI analysis of genotype‐by‐environment data. Crop Science. 2007;47(2):643–53. doi: 10.2135/cropsci2006.06.0374

[17] Becker HC, Léon J. Stability analysis in plant breeding. Plant Breeding. 1988;101(1):1–23. doi: 10.1111/j.1439-0523.1988.tb00261.x

[18] Hudson AI, Odell SG, Dubreuil P, Tixier M-H, Praud S, Runcie DE, et al. Analysis of genotype-by-environment interactions in a maize mapping population. G3 (Bethesda). 2022;12(3):jkac013. doi: 10.1093/g3journal/jkac013 35134181 PMC8895993

[19] Hammami MDE, Álvarez-Iglesias L, Revilla P. Evaluation of the Spanish maize landrace core collection for drought tolerance. Ital J Agron. 2025;20(4):100055.

[20] Garoma B, Alamirew S, Tilahun B. Genotype × environment interaction and grain yield stability of maize (Zea mays L.) hybrids tested in multi-environment trials. 2020.

[21] Westhues CC, Mahone GS, da Silva S, Thorwarth P, Schmidt M, Richter J-C, et al. Prediction of maize phenotypic traits with genomic and environmental predictors using gradient boosting frameworks. Front Plant Sci. 2021;12:699589. doi: 10.3389/fpls.2021.699589 34880880 PMC8647909

[22] Yue H, Olivoto T, Bu J, Li J, Wei J, Xie J, et al. Multi-trait selection for mean performance and stability of maize hybrids in mega-environments delineated using envirotyping techniques. Front Plant Sci. 2022;13:1030521. doi: 10.3389/fpls.2022.1030521 36452111 PMC9702090

[23] Hammami MDE, Lasram A, Kthiri Z, Boukef S, Hamada W, Revilla P, et al. Assessing yield and productivity gaps in Tunisian maize cropping system. Agronomy. 2025;15(2):331. doi: 10.3390/agronomy15020331

[24] Hammami MDE, Madur D, Kthiri Z, Galaretto A, Nicolas SD, Charcosset A, et al. Phylogenetic relationships and genetic diversity of Tunisian maize landraces. PLoS One. 2025;20(1):e0316185. doi: 10.1371/journal.pone.0316185 39841663 PMC11753626

[25] Kthiri Z. Drought tolerance assessment in maize hybrids: morphophysiological and biochemical characterization. SABRAO J Brees Genet. 2024;56(6):2341–50. doi: 10.54910/sabrao2024.56.6.15

[26] Allen RG, Pereira LS, Raes D, Smith M. Crop evapotranspiration: guidelines for computing crop water requirements. Rome: FAO. 1998.

[27] Olivoto T. Metan: Multi environment trials analysis. 2023.

[28] George H. Hargreaves, Zohrab A. Samani. Reference Crop Evapotranspiration from Temperature. Applied Eng Agriculture. 1985;1(2):96–9. doi: 10.13031/2013.26773

[29] SAS Institute Inc. SAS OnlineDoc, version 9. Cary (NC): SAS Institute Inc. 2002.

[30] Posit T. RStudio: Integrated Development Environment for R. Boston (MA): Posit Software, PBC. 2024.

[31] Tonk FA, Ilker E, Tosun M. Evaluation of genotype x environment interactions in maize hybrids using GGE biplot analysis. Crop Breed Appl Biotechnol. 2011;11(1):01–9. doi: 10.1590/s1984-70332011000100001

[32] Kolde R. pheatmap: Pretty Heatmaps. 2010.

[33] Kamel A, Abonazel MR. A Simple Introduction to regression modeling using R. Computational Journal of Mathematical and Statistical Sciences. 2023;2(1):52–79. doi: 10.21608/cjmss.2023.189834.1002

[34] Kolde R. pheatmap: Pretty Heatmaps. CRAN: Contributed Packages. The R Foundation. 2010. doi: 10.32614/cran.package.pheatmap

[35] Denis JB. Analyse de régression factorielle. Biom-Praximetrie. 1980;20:1–34.

[36] Kebede G, Worku W, Feyissa F, Jifar H. Genotype by environment interaction and stability analysis for selection of superior fodder yield performing oat (Avena sativa L.) genotypes using GGE biplot in Ethiopia. Ecol Genet Genomics. 2023;28:100192. doi: 10.1002/jsfa.6703

[37] Singamsetti A, Shahi JP, Zaidi PH, Seetharam K, Vinayan MT, Kumar M, et al. Genotype × environment interaction and selection of maize (Zea mays L.) hybrids across moisture regimes. Field Crops Research. 2021;270:108224. doi: 10.1016/j.fcr.2021.108224

[38] Silva PC, Sánchez AC, Opazo MA, Mardones LA, Acevedo EA. Grain yield, anthesis-silking interval, and phenotypic plasticity in response to changing environments: evaluation in temperate maize hybrids. Field Crops Research. 2022;285:108583. doi: 10.1016/j.fcr.2022.108583

[39] Nelimor C, Badu-Apraku B, Tetteh AY, N’guetta ASP. Assessment of genetic diversity for drought, heat and combined drought and heat stress tolerance in early maturing maize landraces. Plants (Basel). 2019;8(11):518. doi: 10.3390/plants8110518 31744251 PMC6918211

[40] Zaidi PH, Thayil Vinayan M, Nair SK, Kuchanur PH, Kumar R, Bir Singh S, et al. Heat-tolerant maize for rainfed hot, dry environments in the lowland tropics: From breeding to improved seed delivery. The Crop Journal. 2023;11(4):986–1000. doi: 10.1016/j.cj.2023.06.008

[41] Lv X, Yao Q, Mao F, Liu M, Wang Y, Wang X, et al. Heat stress and sexual reproduction in maize: unveiling the most pivotal factors and the greatest opportunities. J Exp Bot. 2024;75(14):4219–43. doi: 10.1093/jxb/erad506 38183327

[42] Li Y, Zhang P, Sheng W, Zhang Z, Rose RJ, Song Y. Securing maize reproductive success under drought stress by harnessing CO2 fertilization for greater productivity. Front Plant Sci. 2023;14:1221095. doi: 10.3389/fpls.2023.1221095 37860252 PMC10582713

[43] Worku M, Makumbi D, Beyene Y, Das B, Mugo S, Pixley K, et al. Grain yield performance and flowering synchrony of CIMMYT’s tropical maize (Zea mays L.) parental inbred lines and single crosses. Euphytica. 2016;211(3):395–409. doi: 10.1007/s10681-016-1758-3

[44] Troyer AF. Background of U.S. hybrid corn II. Crop Science. 2004;44(2):370–80. doi: 10.2135/cropsci2004.3700

[45] Assefa BT, Chamberlin J, Reidsma P, Silva JV, van Ittersum MK. Unravelling the variability and causes of smallholder maize yield gaps in Ethiopia. Food Sec. 2019;12(1):83–103. doi: 10.1007/s12571-019-00981-4

[46] Araus JL, Serret MD, Edmeades GO. Phenotyping maize for adaptation to drought. Front Physiol. 2012;3:305. doi: 10.3389/fphys.2012.00305 22934056 PMC3429076

[47] Wills DM, Whipple CJ, Takuno S, Kursel LE, Shannon LM, Ross-Ibarra J, et al. From many, one: genetic control of prolificacy during maize domestication. PLoS Genet. 2013;9(6):e1003604. doi: 10.1371/journal.pgen.1003604 23825971 PMC3694832

[48] Soto-Gómez D, Pérez-Rodríguez P. Sustainable agriculture through perennial grains: Wheat, rice, maize, and other species. A review. Agriculture, Ecosystems & Environment. 2022;325:107747. doi: 10.1016/j.agee.2021.107747

[49] Wang N, Liu Q, Ming B, Shang W, Zhao X, Wang X, et al. Impacts of heat stress around flowering on growth and development dynamic of maize (Zea mays L.) ear and yield formation. Plants (Basel). 2022;11(24):3515. doi: 10.3390/plants11243515 36559627 PMC9787957

[50] Niu S, Yu L, Li J, Qu L, Wang Z, Li G, et al. Effect of high temperature on maize yield and grain components: a meta-analysis. Sci Total Environ. 2024;952:175898. doi: 10.1016/j.scitotenv.2024.175898 39222820

