# Supplementary figures and images for: Genotypic variability of Tunisian maize landraces: A valuable genetic resource to mitigate drought and heat stress in the Mediterranean basin
Source: PLoS One. 2025 Dec 16;20(12):e0338577. doi: 10.1371/journal.pone.0338577 (PMC12707654; doi:10.1371/journal.pone.0338577)

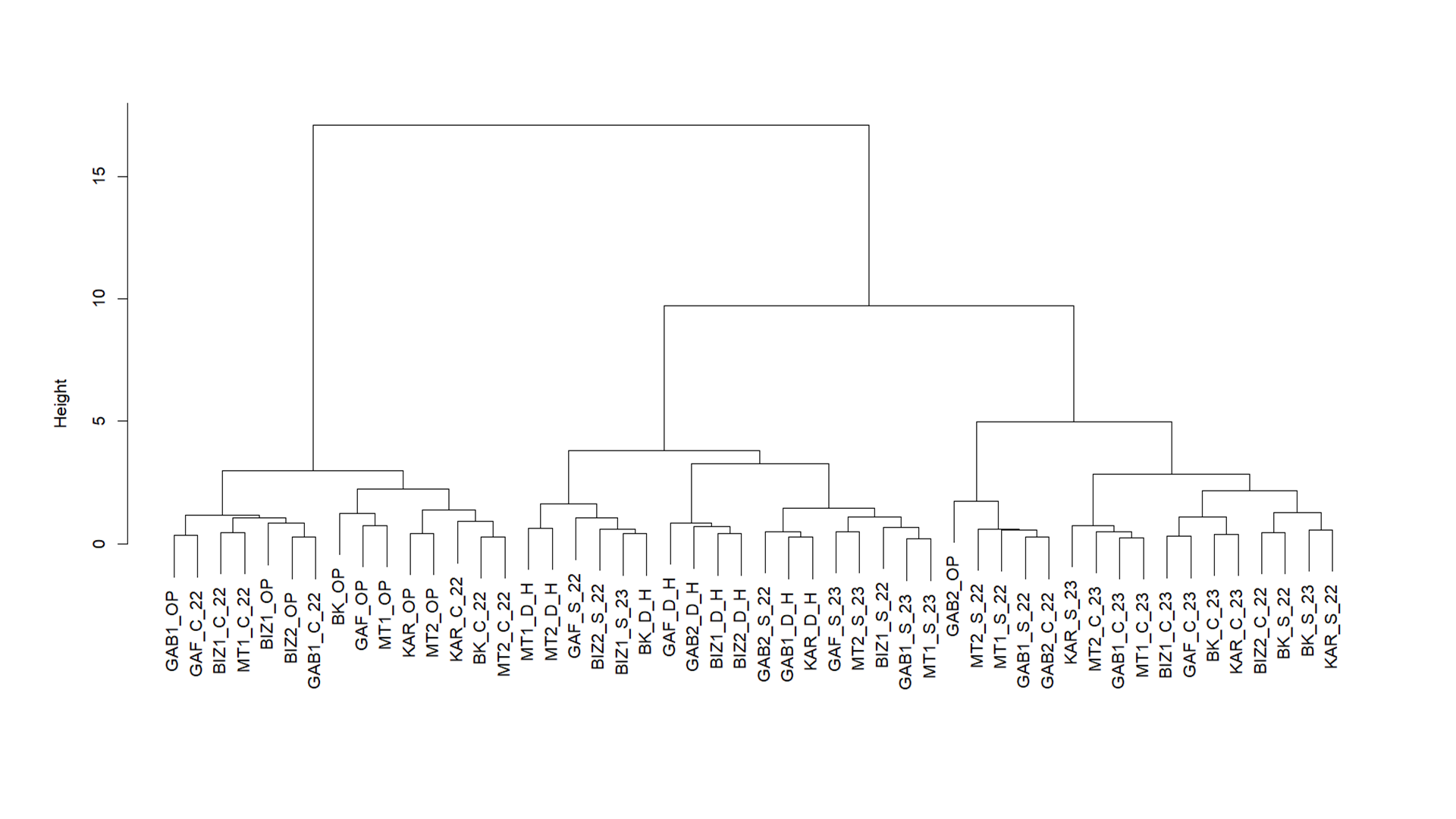

Supplement: S1 Fig — (TIF) [file pone.0338577.s001.tif]

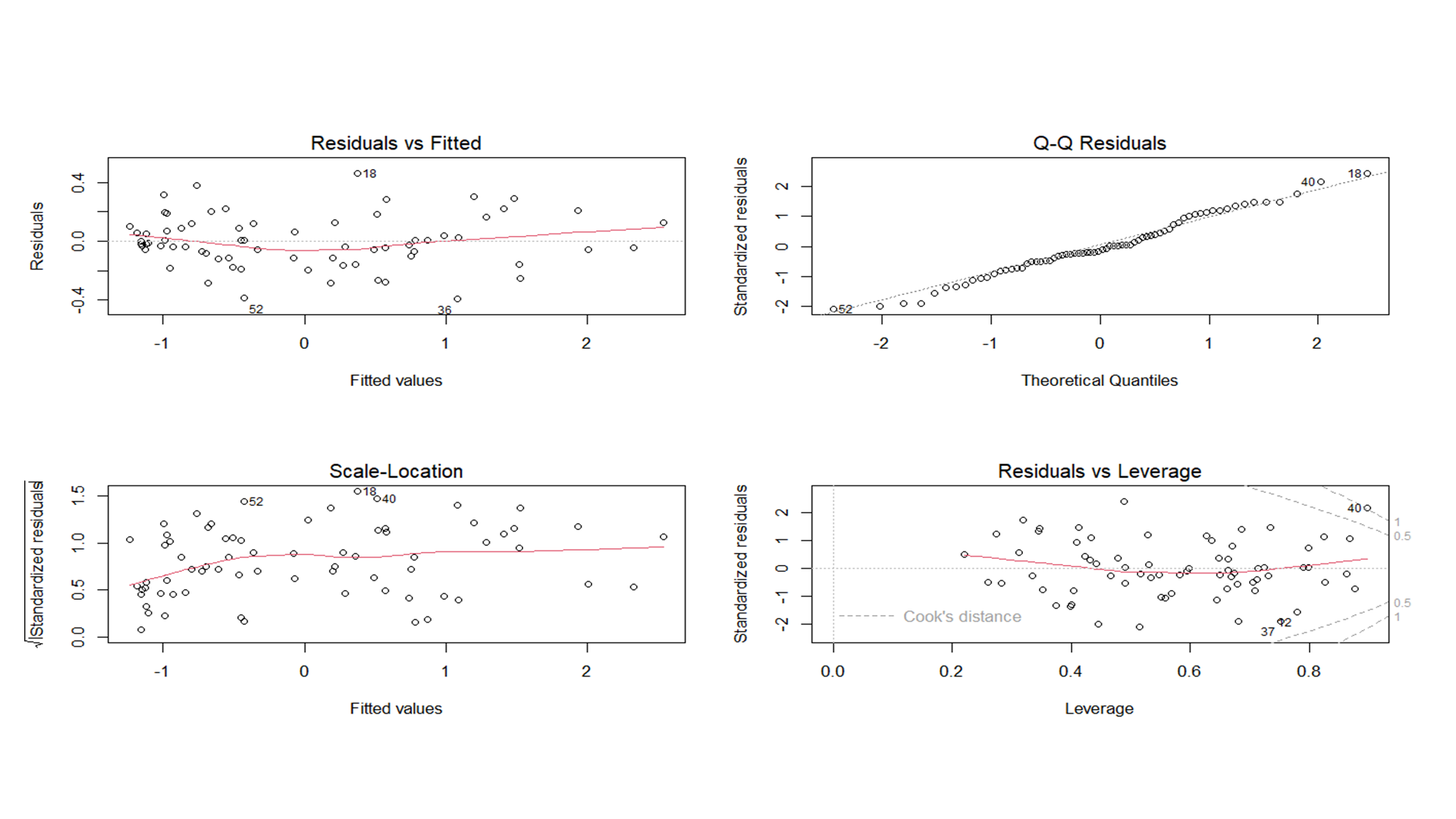

Supplement: S2 Fig — (TIF) [file pone.0338577.s002.tif]
