# Supplementary material for: Genotypic variability of Tunisian maize landraces: A valuable genetic resource to mitigate drought and heat stress in the Mediterranean basin
Source: PLoS One. 2025 Dec 16;20(12):e0338577. doi: 10.1371/journal.pone.0338577 (PMC12707654; doi:10.1371/journal.pone.0338577)
Supplement: Attachment — Submitted filename: Reviewers report.docx [file pone.0338577.s005.docx]

**Reviewer’s Report**

The manuscript titled *"Genotypic variability of Tunisian maize landraces: A Valuable Genetic Resource to Mitigate Drought and Heat Stress in the Mediterranean Basin"* addresses a highly relevant and timely topic in the context of climate change and sustainable agriculture. By evaluating the performance of nine Tunisian maize landraces under diverse environmental conditions, including combined drought and heat stress, the study contributes valuable insights into genotype × environment interactions and stress adaptation mechanisms. The use of multi-environment trials, factorial regression, and trait-environment correlation analysis strengthens the scientific rigor and practical relevance of the research. The identification of promising landraces such as BK, KAR, and MT2 adds breeding value to the work, particularly for developing resilient cultivars suited to arid and semi-arid regions. However, several methodological and interpretational limitations need to be addressed to enhance the clarity, depth, and reproducibility of the study.

**# Major issues**

**1. Experimental Design Issues**

- Unbalanced Environment Representation:
  - Only Mornag had both control and drought stress environments across two years.
  - Sousse (optimal) and Gabes (drought + heat) were tested in only one year each introduces unbalanced data and makes statistical modeling more complex.
- Lack of Replication Across Years:
  - No repeated testing of optimal and drought+heat environments across multiple years → limits ability to separate year vs. environment effects.
- Single-Year Unique Stress (Gabes):
  - Drought + heat environment in Gabes occurred only once; cannot distinguish whether genotype performance is due to stress or year effect.

Clarify this imbalance in the manuscript and you may consider restricting some analyses (e.g., ANOVA, G×E models) to Mornag only, where more balanced replication exists. Use exploratory analyses (e.g., PCA or clustering) for all environments.

Additional concern: Different environments in different years

This adds complexity:

- Year-to-year variation (rainfall, temperature, pests) can mask or mimic stress responses.
- You need to treat year as a factor in your model, but if each environment = one year = one location, you cannot separate these effects.

Unclear total number of entries per block

- With 9 landraces and 5 checks, and 5 blocks:
  - Are all 9 landraces planted once across the trial?
- In a typical augmented design, test genotypes (landraces here) are unreplicated, while checks are replicated.
- Need clarity on how 9 landraces were spread over 5 blocks. With 11 rows per block and 5 check rows, only 6 rows are left for test genotypes — but there are 9 landraces in total. This suggests:
  - Some blocks might have had 2 landraces, others had 3, or they used more than 5 blocks.

**2. Statistical Modeling Limitations**

- Use of Augmented Design:
  - Augmented design is suitable for unreplicated genotypes but requires appropriate statistical corrections (e.g., adjusted means).
  - Block and check structure must be modeled carefully; unclear whether proper error estimation is ensured.
- GGE Biplot Usage:
  - While useful, GGE biplot assumes balanced and replicated environments. Using it with unbalanced data (e.g., unique environments like Gabes or Sousse) may bias interpretation.
- No Inclusion of “Year” as a Factor:
  - Year should be modeled as a random or fixed effect, especially if the same site is tested over years. Ignoring it inflates error terms and biases G×E estimation.

While the augmented design is appropriate for unreplicated landraces, error control depends heavily on check performance and block correction. Inadequate modeling may inflate error or bias genotype effects. Use adjusted means (e.g., using mixed models or method of Federer) before conducting any further analysis (e.g., PCA, GGE, heatmaps). Use GGE biplot for exploratory insight only. For rigorous G×E analysis, apply tools like AMMI or linear mixed models on the balanced subset (e.g., Mornag). Report this limitation clearly. Model year as a factor in ANOVA or mixed models where applicable. Clearly mention this limitation when interpreting genotype × environment interaction (G×E).

Block size and error estimation may be borderline

- With only 5 blocks and 5 replicated checks:
  - Each check is replicated only 5 times,
  - This may give a low degrees of freedom (DF) for the error term,
  - This affects the reliability of ANOVA, especially if checks are not consistent across blocks.

*Acceptable*, but limited power for detecting small differences.

Unclear Justification for Heatmap Use

- Use of heatmaps for genotypes and environments is fine visually but can be misleading without statistical backing due to unbalanced data.

Supplement heatmaps with clustering or PCA for robustness.

**3. Analytical Scope Limitations**

- Mixing Different Stress Types:
  - Combining single-year optimal/drought+heat with multi-year drought trials may obscure true G×E signals and reduce model power.

Avoid over-interpreting results from single-season locations. Use seasonal replication (Mornag) for robust inference; present other data as supplemental.

**# Other important issues (not major but needed to be addressed properly)**

**Abstract**

- Add experimental design info in abstract. No info on replications, plot size, or statistical rigor besides factorial regression. Briefly mention design type (e.g., RCBD with replications) to support validity.
- Line 37: Drought and heat stress…. This line should go later
- Keep consistency in estimates/naming i.e., hydric deficit and water deficit
- No yield ranges, ASI values, or grain weight metrics are included. Include a few key metrics to quantify performance differences (e.g., % yield reduction under stress).
- “Drought and heat stress” are combined in analysis without clear distinction. Clarify whether these stresses occurred independently or in combination and how that was addressed analytically.
- The term “possible stress tolerance mechanisms” is used, but no mechanism is clearly defined. Mention at least one inferred mechanism (e.g., shorter ASI, high TGW under stress).
- It’s not immediately clear whether these are traditional landraces, improved cultivars, or selections. Specify the genetic background to highlight their breeding relevance.
- GAF is mentioned for stable performance but lacks interpretation. Indicate why stable performance under moderate stress is valuable (e.g., for marginal zones).

**M&M**

- Line 106-107: “Each block consisted of 11 rows and included five genotypes per row.”
- Line 122-123: you mentioned frequent heat waves for the Gabes location but the temperature around 35 C. Need explanation
- Mean data of environmental covariates for different locations were not present in the manuscript which is needed.
- Analyzed 10 traits, out of which 5 traits were presented in details environment-wise and others were mean over six environments. Any specific reason? Need clarification. in details environment-wise data required to interpret the variations across the environments specially traits like ASI.
- Line 196: correct the citation style
- Line 196 and line 210: same software RStudio but different citation number
- Spacing Assumption:
  - Claimed plant density of 60,000 plants/ha is only valid if thinning is done precisely.
  - Spacing of 70 cm × 25 cm with 12 hills/row matches this density, but depends on row length and number — needs explicit validation.

Provide detailed field layout and validate plant density calculations. Include actual stand counts if possible.

Data Integrity / Interpretation Concerns:

- Rainfall Variation Doubts:
  - You noted rainfall differences were not substantial, yet the environment was classified as “drought” this might be misleading unless supported by soil moisture or crop stress data.
- Control vs. Optimal Definition Ambiguity:
  - The distinction between “control” and “optimal” seems unclear if climatic conditions were similar — might raise reviewer concerns about justification for separate environmental categories.

Justify environment classification. Alternatively, consider merging or reclassifying environments based on similarity.

**Results**

- Although the ANOVA shows significant G×E interactions for some traits (DT, DS, PH, 1000GW, GYP), the strength and practical implications of these interactions are not quantified or deeply interpreted. Include interaction plots or variance component breakdowns to illustrate the contribution and pattern of G×E effects more clearly.
- Heatmap interpretations are largely qualitative and based on color intensity, which may be subjective. Supplement the heatmap findings with numerical cluster validation methods (e.g., silhouette scores or hierarchical clustering dendrograms) to substantiate clustering.
- Genotypic stability is inferred from proximity to the origin in GGE biplots without using a defined stability index. Calculate and report numerical stability parameters (e.g., Shukla’s stability variance or AMMI stability index) to confirm visual assessments.
- The clusters (A, B, C) identified in the heatmap are not quantitatively validated or connected to broader genetic groupings (e.g., landrace origin, maturity group). Conduct a PCA or clustering analysis based on both phenotypic and environmental responses to support the classification.
- Genotype performance is described visually, but no tables with numerical rankings or genotype × environment scores are provided. Add a supplementary table showing PC1 and PC2 scores or genotype rankings across environments for transparency.
- While factorial regression results are discussed, there's no R², AIC, BIC, or other model fitness indicators reported. Include model fit statistics and possibly residual plots to validate the regression model.
- The results briefly mention that some traits had no significant ENV × GEN effects but don’t report which traits or data. Clarify which traits were not significantly affected by ENV × GEN and provide their data (possibly in a supplementary table).
- Some data are missing in the mean table. Reason behind it and how it was handled during the analysis.

**Discussion**

- The text is dense and often uses long, complex sentences that may reduce readability. Simplify sentence structures and break down long paragraphs for better clarity and reader engagement.
- Significant interaction terms (e.g., PH × DHC, NE × DAY>40) are reported but not biologically contextualized. Briefly explain the biological implications of these interactions (e.g., why PH × DHC has a negative effect) in the results or discussion.
- Factorial regression and GGE biplot results are mentioned, but no statistics (e.g., F-values, R², significance levels) are discussed. Include key statistical metrics to substantiate claims and support interpretations.
- Previous studies citations like [23], [37], [39], etc., and others are used frequently but are not always well integrated into the discussion. Briefly explain the relevance of each cited study and how it supports or contrasts with the findings.
- The discussion spans multiple traits and interactions without summarizing the most impactful results. Add a concluding paragraph that succinctly recaps the top-performing genotypes and most significant trait–environment interactions.
- While the associations/connections between traits and yield are described, the underlying physiological or molecular mechanisms are not discussed. Provide hypotheses or references to explain mechanisms (e.g., why shorter plants perform better under stress).
- Although high-performing landraces are identified, the discussion lacks clear breeding strategy recommendations. Include concrete implications for future breeding efforts, such as which traits to prioritize and potential cross combinations.
- Some traits like ASI and 1000GW are discussed in depth, while others like NGP or AB get little attention. Ensure all analyzed traits receive proportionate discussion, especially those contributing significantly to yield variability.
- Statements about genetic similarity to reference populations (e.g., Reid Yellow Dent) are broad. Include metrics or PCA/phylogenetic figures to substantiate claims of genetic similarity and diversity.
- The ending paragraph is abrupt and doesn’t effectively summarize or transition to conclusions. Add a transition to the conclusion emphasizing the relevance of findings to climate-resilient maize production.

**Conclusion**

- The conclusion makes general claims (e.g., “better yields,” “significant tolerance”) without citing quantitative results or specific statistical outcomes. Include one or two key figures or percentage improvements in yield or ASI to strengthen the claims.
- It mentions “main effect of drought stress” as a limitation but does not clearly distinguish between heat and drought or how their interaction was handled. Clarify whether factorial designs or separate stress-only conditions were used and recommend controlled drought-only trials for future work.
- The only limitation mentioned is the drought-heat overlap, with no reference to possible experimental design constraints, sample size, or genotypic variability limitations. Add 1–2 more concrete limitations—e.g., lack of molecular validation, absence of root traits or physiology-based assessments, or short-term field evaluation—and how future work could address them.
- The conclusion touches briefly on practical applications but lacks depth in addressing policy, breeding program integration, or climate resilience strategies. Expand with a statement like: “These findings can guide national maize breeding programs aiming to develop climate-resilient varieties tailored for North African drylands.”
- Phrases such as “drought tolerance,” “adaptability to stressed conditions,” and “resilience to climatic changes” are repeated without variation or deeper insight. Rephrase and diversify wording. For example:
  - “...resilient performance under compound abiotic stresses...”
  - “...robust reproductive synchronization despite thermal and hydric challenges...”
- While the conclusion mentions potential for breeding, it doesn’t outline next steps or specific breeding strategies. Add a forward-looking statement like:
  “Future work should focus on integrating these landraces into pre-breeding pipelines, leveraging marker-assisted selection or genomic prediction tools to accelerate drought-resilience trait introgression.”
- The final sentence is too generic (“showing more adaptability to stressed conditions”), lacking a strong, memorable message. Strengthen the closing line to reflect a clear takeaway:
  *“Overall, this study lays the groundwork for leveraging native Tunisian maize diversity to safeguard future maize production under increasing climatic pressures.”*

**References**

Some inconsistencies were observed in the bibliography formatting. Please be in line with the journals specific format. Double check the reference section.

**Tables and figures**

Table numbers started with “S” which generally specifies for supplementary tables. Need correction.

Figures quality was not up to the mark. Must be improved
