# Supplementary material for: Genotypic variability of Tunisian maize landraces: A valuable genetic resource to mitigate drought and heat stress in the Mediterranean basin
Source: PLoS One. 2025 Dec 16;20(12):e0338577. doi: 10.1371/journal.pone.0338577 (PMC12707654; doi:10.1371/journal.pone.0338577)
Supplement: Attachment — Submitted filename: Response Letter to Reviewers.docx [file pone.0338577.s006.docx]

**Response Letter to Reviewer**

**Reviewer Comment:**

The manuscript titled *"Genotypic variability of Tunisian maize landraces: A Valuable Genetic Resource to Mitigate Drought and Heat Stress in the Mediterranean Basin"* addresses a highly relevant and timely topic in the context of climate change and sustainable agriculture. By evaluating the performance of nine Tunisian maize landraces under diverse environmental conditions, including combined drought and heat stress, the study contributes valuable insights into genotype × environment interactions and stress adaptation mechanisms. The use of multi-environment trials, factorial regression, and trait-environment correlation analysis strengthens the scientific rigor and practical relevance of the research. The identification of promising landraces such as BK, KAR, and MT2 adds breeding value to the work, particularly for developing resilient cultivars suited to arid and semi-arid regions. However, several methodological and interpretational limitations need to be addressed to enhance the clarity, depth, and reproducibility of the study.

**# Major issues**

**1. Experimental Design Issues**

- Unbalanced Environment Representation:
  - Only Mornag had both control and drought stress environments across two years.
  - Sousse (optimal) and Gabes (drought + heat) were tested in only one year each introduces unbalanced data and makes statistical modeling more complex.
- Lack of Replication Across Years:
  - No repeated testing of optimal and drought+heat environments across multiple years → limits ability to separate year vs. environment effects.
- Single-Year Unique Stress (Gabes):
  - Drought + heat environment in Gabes occurred only once; cannot distinguish whether genotype performance is due to stress or year effect.

Clarify this imbalance in the manuscript and you may consider restricting some analyses (e.g., ANOVA, G×E models) to Mornag only, where more balanced replication exists. Use exploratory analyses (e.g., PCA or clustering) for all environments.

**Response to Reviewer:**

We appreciate the reviewer’s observations regarding the unbalanced representation of environments and the lack of replication across years. However, we would like to clarify that the main objective of our study is not to compare specific geographic locations, but rather to evaluate the performance of Mediterranean maize landraces under distinct cropping conditions, which were intentionally created or naturally occurred in each site.

More specifically:

In Mornag, the experiment was conducted in both 2022 and 2023 under two water regimes: moderate water stress with coinciding heat waves (S_22 and S_23), and full irrigation with heat waves (C_22 and C_23). This allowed us to evaluate genotype performance under reproducible, yet contrasting, stress conditions.

• The optimal environment (Sousse, 2022) and the combined drought and heat stress (Gabes, 2022) were included to broaden the range of Mediterranean growing conditions, even if they were tested in only one year. These environments represent important abiotic stress scenarios in Mediterranean agriculture.

Therefore, the intention was not to analyze regional or year effects, but rather to understand genotypic responses across six distinct Mediterranean cropping conditions, which we treated as fixed environments in the statistical analysis. This aligns with the goal of identifying landraces with superior performance under specific abiotic stress combinations.

To clarify this in the manuscript, we have added Table 4: Agro-Climatic Description and Abiotic Stress Levels of Experimental Environments, which provides a detailed characterization of each environment and justifies their inclusion in the study.

**Reviewer Comment:**

Additional concern: Different environments in different years

This adds complexity:

- Year-to-year variation (rainfall, temperature, pests) can mask or mimic stress responses.
- You need to treat year as a factor in your model, but if each environment = one year = one location, you cannot separate these effects.

**Response to Reviewer:**

We appreciate the reviewer’s concern regarding the potential confounding between year, location, and environment. However, we would like to clarify that in this study, our aim was not to analyze year effects, but rather to create and capture a wider range of Mediterranean cropping conditions, including various combinations of water regimes and thermal stress.

The two additional environments in 2023 (C_23 and S_23) were not introduced to study year-to-year variation, but rather to increase environmental diversity and enrich the evaluation of landrace performance under relevant stress scenarios. Therefore, we do not consider it necessary to include “year” as a factor in the model, since the environments themselves defined by their agro-climatic characteristics and stress levels are the primary focus of our analysis.

The observed imbalance in environmental representation is due to the fact that the Mornag locality was the only site where we were able to conduct trials under both control and drought conditions across two consecutive years. We considered it valuable to have at least one location with replicated trials under both stress and non-stress conditions, in order to strengthen the reliability of comparisons in these two key environments. Moreover, in our model, environments were treated as fixed factors, and our goal was to compare and identify landraces that perform well under six distinct environmental scenarios, rather than to study temporal effects. This has been clarified in the revised version of the manuscript, along with the inclusion of Table 4, which provides a detailed agro-climatic description of all environments to support this interpretation.

**Reviewer Comment:**

Unclear total number of entries per block

- With 9 landraces and 5 checks, and 5 blocks:
  - Are all 9 landraces planted once across the trial?
- In a typical augmented design, test genotypes (landraces here) are unreplicated, while checks are replicated.
- Need clarity on how 9 landraces were spread over 5 blocks. With 11 rows per block and 5 check rows, only 6 rows are left for test genotypes — but there are 9 landraces in total. This suggests:
  - Some blocks might have had 2 landraces, others had 3, or they used more than 5 blocks.

**Response to Reviewer:**

We thank the reviewer for pointing out the need for greater clarity regarding the experimental design.

We would like to clarify that the Tunisian landraces analyzed in this study represent a subset (9 entries) of a larger panel of 255 Mediterranean varieties and selections cycles evaluated in the full trial. For the purpose of this manuscript, the analysis focused specifically on the 9 Tunisian landraces, as the objective was to assess their performance under different Mediterranean stress conditions.

The trial was conducted using an augmented design over two consecutive years. In the first year, the design included 5 blocks with 54 plots per block. Of these, 3 plots in each block were allocated to check varieties, which were replicated across all blocks, while the remaining 51 plots were assigned to unreplicated landraces. In the second year, the augmented design consisted of 55 plots per block, with 4 plots assigned to replicated check varieties and 51 plots allocated to unreplicated landraces. The remaining plots in both years were used to randomly assign the landraces across blocks.

Since the 9 Tunisian landraces were not replicated, their distribution varied slightly across environments. For example, in environment C_22, the distribution was as follows:

Block 1: BIZ1, BK

Block 2: GAB2, GAF

Block 3: KAR, GAB1

Block 4: BIZ2, MT1

Block 5: MT2

In other environments, a different random assignment of landraces was used, always respecting the augmented design structure.

We have revised and clarified the Field Trials section in the manuscript to better reflect this structure and improve transparency for the reader.

**Reviewer Comment:**

**2. Statistical Modeling Limitations**

- Use of Augmented Design:
  - Augmented design is suitable for unreplicated genotypes but requires appropriate statistical corrections (e.g., adjusted means).
  - Block and check structure must be modeled carefully; unclear whether proper error estimation is ensured.

**Response to Reviewer:**

We appreciate the reviewer’s comment regarding the use of the augmented design. In our study, this design was chosen due to the large number of landraces evaluated, which made full replication logistically unfeasible. To ensure reliable error estimation and accurate adjustment of means, we included replicated checks in each of the five blocks. These checks enabled the estimation of block effects and error variance, which were used to adjust the means of the unreplicated landraces. Least Squares Means (LS means) were calculated using the Proc Mixed procedure in SAS, treating genotypes and environments as fixed effects while blocks nested within environments as Random effect. To compare the LS means of the landraces we calculate the Least Significant Difference (LSD) at P < 0.05 using the checks. Furthermore, for traits showing a significant genotype × environment interaction, separate analyses of variance were conducted within each environment while for traits without significant interaction, the LSD was derived from the combined analysis across environments.

**Reviewer Comment:**

- GGE Biplot Usage:
  - While useful, GGE biplot assumes balanced and replicated environments. Using it with unbalanced data (e.g., unique environments like Gabes or Sousse) may bias interpretation.

**Response to Reviewer:**

We thank the reviewer for this important comment. In our study, GGE biplot analysis was used to evaluate genotype × environment interactions, focusing on both the "Mean vs. Stability" and "Which-won-where" views to identify superior landraces under varying conditions. Our objective was not to compare specific locations, but rather to assess genotypic performance across distinct agronomic conditions that represent contrasting Mediterranean stress scenarios. To address the unbalanced nature of the data, LS means (Least Squares Means) adjusted per block were used as input for the GGE biplot analysis, ensuring a more accurate comparison despite the lack of full replication.

**Reviewer Comment:**

- No Inclusion of “Year” as a Factor:
  - Year should be modeled as a random or fixed effect, especially if the same site is tested over years. Ignoring it inflates error terms and biases G×E estimation.

**Response to Reviewer:**

We thank the reviewer for highlighting this important point. The primary objective of our study was to evaluate the performance and stability of Mediterranean maize landraces under contrasting agro-environmental conditions.

While the effect of “year” was not modeled separately, each environment (C_22, C_23, S_22, S_23, OP, D_H) was deliberately defined as a unique combination of year × management conditions, representing distinct Mediterranean stress scenarios (such as moderate drought, combined drought and heat, or optimal conditions). Therefore, we treated “environment” as a fixed effect characterizing these specific growing conditions, rather than as a proxy for year or site. The repetition of the trial in 2023 at the same location (Mornag) under both full irrigation and moderate drought (C_23 and S_23) was intended to expand the range of environments, not to assess year effects per se. This design choice allowed us to increase environmental diversity and strengthen genotype evaluation under relevant Mediterranean scenarios.

We acknowledge that this approach does not allow a complete separation of year and environment effects. However, we believe this limitation does not undermine the main objective of the study, which was to compare genotype performance and stability across well-characterized and representative stress environments.

**Reviewer Comment:**

While the augmented design is appropriate for unreplicated landraces, error control depends heavily on check performance and block correction. Inadequate modeling may inflate error or bias genotype effects. Use adjusted means (e.g., using mixed models or method of Federer) before conducting any further analysis (e.g., PCA, GGE, heatmaps). Use GGE biplot for exploratory insight only. For rigorous G×E analysis, apply tools like AMMI or linear mixed models on the balanced subset (e.g., Mornag). Report this limitation clearly. Model year as a factor in ANOVA or mixed models where applicable. Clearly mention this limitation when interpreting genotype × environment interaction (G×E).

Block size and error estimation may be borderline

- With only 5 blocks and 5 replicated checks:
  - Each check is replicated only 5 times,
  - This may give a low degrees of freedom (DF) for the error term,
  - This affects the reliability of ANOVA, especially if checks are not consistent across blocks.

*Acceptable*, but limited power for detecting small differences.

Unclear Justification for Heatmap Use

- Use of heatmaps for genotypes and environments is fine visually but can be misleading without statistical backing due to unbalanced data.

Supplement heatmaps with clustering or PCA for robustness.

**3. Analytical Scope Limitations**

- Mixing Different Stress Types:
  - Combining single-year optimal/drought+heat with multi-year drought trials may obscure true G×E signals and reduce model power.

Avoid over-interpreting results from single-season locations. Use seasonal replication (Mornag) for robust inference; present other data as supplemental.

**Response to Reviewer:**

We thank the reviewer for the detailed and constructive comments regarding the augmented design and subsequent analyses.

As outlined in the revised Materials and Methods section, the experiment was conducted using an augmented design with five blocks. Within each block, 3 plots (in 2022) or 4 plots (in 2023) were allocated to check varieties, which were replicated across all five blocks, ensuring 15 and 20 replicated plots per environment. The remaining plots in each block were used to randomly assign the unreplicated landraces. This design allowed estimation of block effects and error variance using the replicated checks, enabling adjustment of landrace means and valid within-environment comparisons. The Least Squares Means (LS means) were obtained using the Proc Mixed procedure in SAS, treating blocks nested within environments as Random effect and environments and genotypes as fixed effects.

Regarding G×E interaction, we used GGE biplot analysis as an exploratory tool to visualize genotype performance and stability across environments. These environments were not treated merely as years or sites, but as unique combinations of year × water/heat regime (e.g., C_22, S_23, OP, D_H), reflecting distinct Mediterranean stress scenarios. The LS means adjusted per blocks were used as input in the GGE biplot, and we clarify that interpretations are exploratory and should be viewed with this limitation in mind.

Concerning the heatmaps, we agree that unbalanced data can lead to misleading visual interpretations if not appropriately treated. To reinforce the analysis, we conducted a hierarchical clustering of genotypes based on their adjusted LS means adjusted per blocks across environments. These clusters were integrated into the heatmap visualization and are now included in the Supplementary Material (S1 Fig) to support the robustness of the visual analysis.

Lastly, we acknowledge that year effects were not modeled as a separate factor. Instead, "environments" in our study were defined as specific combinations of year × treatment, and were treated as fixed.

**Reviewer Comment:**

**# Other important issues (not major but needed to be addressed properly)**

**Abstract**

- Add experimental design info in abstract. No info on replications, plot size, or statistical rigor besides factorial regression. Briefly mention design type (e.g., RCBD with replications) to support validity.
- Line 37: Drought and heat stress…. This line should go later
- Keep consistency in estimates/naming i.e., hydric deficit and water deficit
- No yield ranges, ASI values, or grain weight metrics are included. Include a few key metrics to quantify performance differences (e.g., % yield reduction under stress).
- “Drought and heat stress” are combined in analysis without clear distinction. Clarify whether these stresses occurred independently or in combination and how that was addressed analytically.
- The term “possible stress tolerance mechanisms” is used, but no mechanism is clearly defined. Mention at least one inferred mechanism (e.g., shorter ASI, high TGW under stress).
- It’s not immediately clear whether these are traditional landraces, improved cultivars, or selections. Specify the genetic background to highlight their breeding relevance.
- GAF is mentioned for stable performance but lacks interpretation. Indicate why stable performance under moderate stress is valuable (e.g., for marginal zones).

**Response to Reviewer:**

We thank the reviewer for these valuable suggestions. We have revised the abstract accordingly by adding key information on the experimental design, including replication and design type to support the study's validity. The positioning of the sentence regarding drought and heat stress has been adjusted for better flow. Terminology has been standardized for consistency (using “water deficit” throughout). We included important quantitative metrics such as yield reductions, ASI values, and grain weight changes to better illustrate performance differences. The combined and overlapping nature of drought and heat stress is now clearly explained. Finally, we clarified the genetic background of the materials, specifying that these are traditional Tunisian landraces to emphasize their breeding relevance. In the previous version, GAF was mentioned in the abstract for its stable performance; however, upon revision, we realized that the statement lacked sufficient context and interpretation. As a result, we decided to remove it from the abstract to maintain clarity and focus on key findings.

**Reviewer Comment:**

**M&M**

- Line 106-107: “Each block consisted of 11 rows and included five genotypes per row.”
- Line 122-123: you mentioned frequent heat waves for the Gabes location but the temperature around 35 C. Need explanation
- Mean data of environmental covariates for different locations were not present in the manuscript which is needed.
- Analyzed 10 traits, out of which 5 traits were presented in details environment-wise and others were mean over six environments. Any specific reason? Need clarification. in details environment-wise data required to interpret the variations across the environments specially traits like ASI.

**Response to Reviewer:**

We thank the reviewer for this insightful comment. Among the ten traits analyzed, detailed environment-wise data were presented only for the five traits that exhibited significant genotype × environment (G×E) interaction, as detected in the combined ANOVA. For the remaining traits, including ASI, AB, NG, and NE no significant G×E interaction was observed. Therefore, we reported the combined means across environments, since evaluating their variation environment by environment would not provide additional meaningful insight in the absence of interaction.

However, in response to the reviewer’s request and to enhance transparency, we have now included the environment-wise means for these traits in a supplementary table (S1 Table)

**Reviewer Comment:**

- Line 196: correct the citation style
- Line 196 and line 210: Same software RStudio but different citation number

**Response to Reviewer:**

Thank you for your observation. We have carefully reviewed the citation style used in the manuscript, especially for software references.

**Reviewer Comment:**

- Spacing Assumption:
  - Claimed plant density of 60,000 plants/ha is only valid if thinning is done precisely.
  - Spacing of 70 cm × 25 cm with 12 hills/row matches this density, but depends on row length and number — needs explicit validation.

**Response to Reviewer:**

We thank the reviewer for pointing out the need for clarification regarding plant spacing and density. We acknowledge that the original explanation was unclear, and we have revised the text accordingly for accuracy.

The genotypes, including landraces and checks, were cultivated in plots measuring three meters in length, with 12 hills per row. Each hill was sown with two seeds. The spacing between plots was 50 cm, and the spacing between hills within the same row was 25 cm. Thinning was performed at the two-leaf stage to leave one plant per hill. This thinning ensured a final plant density of approximately 60,000 plants per hectare.

These corrections have been incorporated into the manuscript to provide a clearer and more precise description of the planting design and density assumptions.

**Reviewer Comment:**

Provide detailed field layout and validate plant density calculations. Include actual stand counts if possible.

Data Integrity / Interpretation Concerns:

- Rainfall Variation Doubts:
  - You noted rainfall differences were not substantial, yet the environment was classified as “drought” this might be misleading unless supported by soil moisture or crop stress data.
- Control vs. Optimal Definition Ambiguity:
  - The distinction between “control” and “optimal” seems unclear if climatic conditions were similar — might raise reviewer concerns about justification for separate environmental categories.

Justify environment classification. Alternatively, consider merging or reclassifying environments based on similarity.

**Response to Reviewer:**

We thank the reviewer for raising these important points concerning the characterization of environmental conditions and the validation of plant density assumptions.

To address the concern regarding rainfall variation and environmental classification, we have included in the revised manuscript Table 4: Agro-Climatic Description and Abiotic Stress Levels of Experimental Environments, which provides comprehensive climate data and derived crop stress indicators for each environment. This table includes minimum and maximum temperatures, rainfall (RA), evapotranspiration (ETP), water deficit (DHC – Hydric Deficit), Water Stress Index (WSI), Anthesis-Silking Interval environment mean (ASI_ENV), and the number of days with temperatures above 40°C (DAY>40), along with a qualitative description of the resulting growth conditions. While rainfall differences between some environments may appear moderate, the integrated stress indicators clearly demonstrate that environments classified as “drought” or “combined heat and drought stress” experienced substantial crop water deficits and physiological stress. For example, severe water deficits (negative DHC), elevated WSI values, extended ASI, and a higher frequency of extreme temperatures (DAY>40) provide quantitative evidence supporting our classification. Thus, the stress categories assigned to the environments are based not only on rainfall but on a combination of agro-climatic and physiological indicators, offering a robust and biologically meaningful basis for environmental characterization. Regarding the distinction between “control” and “optimal” environments, these represent different management and climatic conditions as reflected in the detailed agro-climatic parameters. The "control" environments in 2022 (C_22) and 2023 (C_23) correspond to full irrigation treatments, where water supply was maintained at approximately 90% of field capacity to provide near-optimal soil moisture conditions. However, during these growing seasons, natural heat waves occurred, resulting in episodes of elevated temperatures that imposed moderate heat stress on the maize plants, despite the adequate irrigation.

This situation is supported by our agro-climatic data (Table 4), which shows higher maximum temperatures and increased stress indices during these periods, leading us to classify these control plots as experiencing moderate heat stress conditions rather than truly optimal conditions. Thus, although the irrigation regime aimed to minimize drought stress, the thermal stress component was unavoidable and relevant to the Mediterranean environment scenarios we intended to simulate.

We have clarified this point in the manuscript to avoid confusion and to justify the environmental classification accurately. This characterization is important for interpreting genotype performance under realistic Mediterranean climate challenges, where heat stress often co-occurs with or follows well-irrigated conditions.

**Reviewer Comment:**

**Results**

- Although the ANOVA shows significant G×E interactions for some traits (DT, DS, PH, 1000GW, GYP), the strength and practical implications of these interactions are not quantified or deeply interpreted. Include interaction plots or variance component breakdowns to illustrate the contribution and pattern of G×E effects more clearly.

**Response to Reviewer:**

We thank the reviewer for this relevant suggestion regarding the interpretation of G×E interactions. While we did not present a full variance component breakdown, we acknowledge the importance of better illustrating these interactions. In response to significant G×E interactions observed for traits such as GYP, PH, and 1000GW, we explored genotype performance and stability using the Mean vs. Stability plot and GGE Biplot analysis, which are well-established approaches for visualizing and interpreting genotype responses across environments. These plots allowed us to identify genotypes that combined both high performance and stability under contrasting environmental conditions. Furthermore, to better understand the influence of both genotypic and environmental factors on grain yield (GYP), we conducted a factorial regression analysis, incorporating key genotypic covariates, as well as environmental indices and their interactions. This approach allowed us to quantify how these variables contributed to yield performance under contrasting conditions, providing an alternative and informative interpretation of the G×E patterns.

**Reviewer Comment:**

- Heatmap interpretations are largely qualitative and based on color intensity, which may be subjective. Supplement the heatmap findings with numerical cluster validation methods (e.g., silhouette scores or hierarchical clustering dendrograms) to substantiate clustering.
- Genotypic stability is inferred from proximity to the origin in GGE biplots without using a defined stability index. Calculate and report numerical stability parameters (e.g., Shukla’s stability variance or AMMI stability index) to confirm visual assessments.

**Response to Reviewer:**

We thank the reviewer for the valuable suggestion. Our stability inference is not solely based on the proximity to the origin in the GGE biplots. In addition, we used the **Mean vs. Stability** plot, which is specifically designed to evaluate genotype stability numerically and visually by combining mean performance and stability parameters. We did not calculate Shukla’s stability variance in this study. Instead, for stability analysis, we relied on the Mean vs. Stability plot, which is a widely accepted method to combine mean performance and stability visually and numerically. Regarding AMMI, we chose to use GGE biplots because they are considered more comprehensive and powerful, as GGE biplots capture both genotype main effects and genotype-by-environment interaction effects simultaneously by focusing on principal components that summarize the variation more effectively. This makes GGE biplots particularly suitable for evaluating genotype stability and performance across multiple environments in our context.

**Reviewer Comment:**

- The clusters (A, B, C) identified in the heatmap are not quantitatively validated or connected to broader genetic groupings (e.g., landrace origin, maturity group). Conduct a PCA or clustering analysis based on both phenotypic and environmental responses to support the classification.

**Response to Reviewer:**

We thank the reviewer for this important comment. To support and validate the cluster groupings (A, B, C) identified in the heatmap, we have conducted a clustering analysis based on phenotypic and environmental response data. The clustering analysis was included in the Supplementary Materials (**S1 Figure)**.

**Reviewer Comment:**

- Genotype performance is described visually, but no tables with numerical rankings or genotype × environment scores are provided. Add a supplementary table showing PC1 and PC2 scores or genotype rankings across environments for transparency.

**Response to Reviewer:**

We thank the reviewer for this valuable suggestion. To enhance transparency and provide detailed quantitative information, we have included a supplementary table presenting the PC1 and PC2 scores of each genotype in each environment. This table is available in the Supplementary Materials section (S2 table).

**Reviewer Comment:**

- While factorial regression results are discussed, there's no R², AIC, BIC, or other model fitness indicators reported. Include model fit statistics and possibly residual plots to validate the regression model.

**Response to Reviewer:**

Thank you for requesting additional diagnostics to support the validity of our factorial regression on grain yield. We have generated the four standard residual plots and performed formal tests for normality and homoscedasticity (S2 Figure).

1. **Residuals vs. Fitted Values**
   - The residuals are scattered randomly around zero, with no discernible U‑shape or funnel pattern, indicating that the linearity assumption holds and that variance does not systematically change with the level of the fitted values.
2. **Normal Q–Q Plot**
   - The points lie closely along the 45° reference line, demonstrating that the residuals follow an approximately normal distribution.
3. **Scale–Location Plot**
   - The square‑root of the absolute standardized residuals shows no trend across fitted values (no cone‑shaped pattern), confirming homoscedasticity.
4. **Residuals vs. Leverage**
   - No observations exceed common Cook’s distance thresholds, and there are no high‑leverage points unduly influencing the model.
5. **Formal Tests**
   - **Breusch–Pagan test for heteroscedasticity**: BP = 32.323 (df = 39), p‑value = 0.7665. We fail to reject homoscedasticity.
   - **Shapiro–Wilk test for normality**: W = 0.9881, p‑value = 0.7482. We fail to reject normality of residuals.

Together, these diagnostics confirm that the key assumptions of our factorial regression are met. Consequently, the estimated covariance parameters genotypic effects (α), environmental effects (β), and genotype×environment interaction effects (θ) on grain yield can be interpreted with confidence as reflecting genuine biological variability rather than model artefacts.

**Reviewer Comment:**

- The results briefly mention that some traits had no significant ENV × GEN effects but don’t report which traits or data. Clarify which traits were not significantly affected by ENV × GEN and provide their data (possibly in a supplementary table).

**Response to Reviewer:**

We thank the reviewer for this important remark. In the manuscript, we clarify that certain traits, such as AB, ASI, NE, NG, and NGP, did not show significant G×E interactions based on our ANOVA results. Because no significant interaction was detected for these traits, it was not considered relevant to evaluate genotype performance environment-wise; therefore, we worked with combined data across environments to compare genotype performance. However, for greater transparency, we have now included a supplementary table (S1 Table) presenting the LS means of each genotype under each environment for traits without significant interaction.

**Reviewer Comment:**

- Some data are missing in the mean table. Reason behind it and how it was handled during the analysis.

**Response to Reviewer:**

We thank the reviewer for this observation. Missing data were primarily due to germination failure in some landraces, likely caused by unfavorable environmental or growth conditions. To handle these missing values during analysis, we used the check varieties to estimate the Least Significant Difference (LSD), and calculated least square means (LS means) adjusted per block. These adjusted means were then used as input for the GGE biplot analysis. This method allowed us to manage the unbalanced dataset and ensure reliable estimation and comparison across genotypes.

**Reviewer Comment:**

**Discussion**

- The text is dense and often uses long, complex sentences that may reduce readability. Simplify sentence structures and break down long paragraphs for better clarity and reader engagement.
- Significant interaction terms (e.g., PH × DHC, NE × DAY>40) are reported but not biologically contextualized. Briefly explain the biological implications of these interactions (e.g., why PH × DHC has a negative effect) in the results or discussion.
- Factorial regression and GGE biplot results are mentioned, but no statistics (e.g., F-values, R², significance levels) are discussed. Include key statistical metrics to substantiate claims and support interpretations.
- Previous studies citations like [23], [37], [39], etc., and others are used frequently but are not always well integrated into the discussion. Briefly explain the relevance of each cited study and how it supports or contrasts with the findings.
- The discussion spans multiple traits and interactions without summarizing the most impactful results. Add a concluding paragraph that succinctly recaps the top-performing genotypes and most significant trait–environment interactions.
- While the associations/connections between traits and yield are described, the underlying physiological or molecular mechanisms are not discussed. Provide hypotheses or references to explain mechanisms (e.g., why shorter plants perform better under stress).
- Although high-performing landraces are identified, the discussion lacks clear breeding strategy recommendations. Include concrete implications for future breeding efforts, such as which traits to prioritize and potential cross combinations.
- Some traits like ASI and 1000GW are discussed in depth, while others like NGP or AB get little attention. Ensure all analyzed traits receive proportionate discussion, especially those contributing significantly to yield variability.
- Statements about genetic similarity to reference populations (e.g., Reid Yellow Dent) are broad. Include metrics or PCA/phylogenetic figures to substantiate claims of genetic similarity and diversity.

Hammami et al 2025

- The ending paragraph is abrupt and doesn’t effectively summarize or transition to conclusions. Add a transition to the conclusion emphasizing the relevance of findings to climate-resilient maize production.

**Response to Reviewer:**

We thank the reviewer for these insightful and constructive remarks. In response, we have thoroughly revised and streamlined the manuscript text to improve readability by simplifying sentence structures and breaking down dense paragraphs. We enhanced the sections on factorial regression and GGE biplot by adding key statistical metrics, including the R² value (46.6%) for the factorial regression model, to better substantiate the interpretations. The biological implications of significant interaction terms have been more clearly explained in the discussion. We also improved the integration of cited studies by briefly contextualizing their relevance to our findings and expanded the discussion to cover all analyzed traits proportionately, especially those with significant contributions to yield variability.

Furthermore, a concise concluding paragraph summarizing the top-performing genotypes and key genotype × environment interactions has been added, along with concrete breeding strategy recommendations focusing on prioritized traits and potential crosses.

Regarding the statement about genetic similarity to reference populations like Reid Yellow Dent, we referred to our recent publication (Hammami et al., 2025b, PLOS ONE), which includes detailed PCA and phylogenetic analyses substantiating these claims. The reference is now explicitly cited in the manuscript:

Hammami, M. D. E., Madur, D., Kthiri, Z., Galaretto, A., Nicolas, S. D., Charcosset, A., Combes, V., Karmous, C., & Revilla, P. (2025b). Phylogenetic relationships and genetic diversity of Tunisian maize landraces. PLOS ONE, 20(1), e0316185.

<https://doi.org/10.1371/journal.pone.0316185>.

Finally, we have added a transition paragraph leading into the conclusion that emphasizes the relevance of our findings for developing climate-resilient maize varieties.

**Reviewer Comment:**

**Conclusion**

- The conclusion makes general claims (e.g., “better yields,” “significant tolerance”) without citing quantitative results or specific statistical outcomes. Include one or two key figures or percentage improvements in yield or ASI to strengthen the claims.
- It mentions “main effect of drought stress” as a limitation but does not clearly distinguish between heat and drought or how their interaction was handled. Clarify whether factorial designs or separate stress-only conditions were used and recommend controlled drought-only trials for future work.
- The only limitation mentioned is the drought-heat overlap, with no reference to possible experimental design constraints, sample size, or genotypic variability limitations. Add 1–2 more concrete limitations—e.g., lack of molecular validation, absence of root traits or physiology-based assessments, or short-term field evaluation—and how future work could address them.
- The conclusion touches briefly on practical applications but lacks depth in addressing policy, breeding program integration, or climate resilience strategies. Expand with a statement like: “These findings can guide national maize breeding programs aiming to develop climate-resilient varieties tailored for North African drylands.”
- Phrases such as “drought tolerance,” “adaptability to stressed conditions,” and “resilience to climatic changes” are repeated without variation or deeper insight. Rephrase and diversify wording. For example:
  - “...resilient performance under compound abiotic stresses...”
  - “...robust reproductive synchronization despite thermal and hydric challenges...”
- While the conclusion mentions potential for breeding, it doesn’t outline next steps or specific breeding strategies. Add a forward-looking statement like:
  “Future work should focus on integrating these landraces into pre-breeding pipelines, leveraging marker-assisted selection or genomic prediction tools to accelerate drought-resilience trait introgression.”
- The final sentence is too generic (“showing more adaptability to stressed conditions”), lacking a strong, memorable message. Strengthen the closing line to reflect a clear takeaway:
  *“Overall, this study lays the groundwork for leveraging native Tunisian maize diversity to safeguard future maize production under increasing climatic pressures.”*

**Response to Reviewer:**

We thank the reviewer for the valuable comments and suggestions. In response, we have carefully revised and reformulated the conclusion to address your remarks. The revised conclusion now includes specific quantitative results and statistical outcomes, such as the percentage reductions in plant height and grain yield under combined drought and heat stress, to substantiate our claims of genotype adaptation and tolerance.

We clarified the limitation regarding the overlap of drought and heat stress, explaining that it constrains the ability to isolate the effects of each stress individually. We also highlighted the need for future controlled trials focusing separately on drought and heat stresses, alongside the assessment of root traits, physiological indicators, and molecular markers to better understand underlying adaptation mechanisms.

Furthermore, we expanded the practical implications by specifying key traits (high grain yield, short ASI, high grain number, moderate plant height and grain weight) to prioritize in breeding for climate resilience. We emphasized the promise of genotypes BK, KAR, and MT2 as donor lines for pre-breeding and the potential integration of these landraces into marker-assisted or genomic selection pipelines.

Finally, the conclusion now better reflects the broader significance of Tunisian maize genetic diversity as a valuable resource for breeding resilient cultivars suited to Mediterranean drylands and other stress-prone regions, with a strengthened forward-looking perspective.

We trust these improvements adequately address your concerns and enhance the clarity and impact of our manuscript’s conclusion.

**Reviewer Comment:**

**References**

Some inconsistencies were observed in the bibliography formatting. Please be in line with the journals specific format. Double check the reference section.

**Response to Reviewer:**

We thank the reviewer for pointing this out. The reference list has been carefully revised to ensure full compliance with the journal’s formatting guidelines.

**Reviewer Comment:**

**Tables and figures**

Table numbers started with “S” which generally specifies for supplementary tables. Need correction.

Figures quality was not up to the mark. Must be improved

**Response to Reviewer:**

We thank the reviewer for these observations. We have reintroduced all figures with improved resolution and visual clarity to enhance readability. Additionally, the table numbers have been corrected—supplementary tables are now properly distinguished from main tables, and all main tables are renumbered according to the journal's guidelines.
