# Supplementary material for: Genotypic variability of Tunisian maize landraces: A valuable genetic resource to mitigate drought and heat stress in the Mediterranean basin
Source: PLoS One. 2025 Dec 16;20(12):e0338577. doi: 10.1371/journal.pone.0338577 (PMC12707654; doi:10.1371/journal.pone.0338577)
Supplement: Attachment — Submitted filename: Reviewer comments_26.8.25.docx [file pone.0338577.s007.docx]

Thank you for submitting the revised manuscript. The revisions have substantially improved clarity, particularly in the results and discussion sections, with better explanation of trait performance under stress. The abstract, tables, and figures are now clearer and more consistent, and key were well-highlighted. A few minor corrections regarding terminology consistency, grammar issues, typo etc., which will further polish the manuscript. Below is my concern which needed to be addressed.

**Major Concern-**

**Introduction:**

- Clarify the research gap Link objectives clearly to the breeding relevance under Mediterranean stress conditions.
- Clearly state the problem statement (heat stress impact in tropical maize) in the last paragraph.
- Include recent references (last 5 years) on heat stress adaptation in maize.
- Streamline background- reduce long historical references and focus on current breeding challenges.

**Materials and Methods:**

- Include exact numbers of plants sampled for each trait and details on checks/controls.
- Specify statistical software/packages used for factorial regression analysis.
- Provide criteria for selecting genotypes (origin, previous evaluation) and explicitly justify why these nine Tunisian landraces were selected.

**Results:**

- Emphasize patterns and biological interpretations rather than raw percentages;
- Factorial regression interpretation should be illustrated schematically for clarity.
- Present key interactions more clearly (focus on PH × DHC, ASI under stress, NE × DAY>40).
- Remove repetitive mentions of the same superior genotypes in multiple subsections-summarize once.
- Add numeric evidence (means, % changes) when describing genotype performance.

**Discussion:**

- Relate observed phenotypic plasticity to physiological mechanisms and breeding implications.
- Highlight relevance beyond Tunisia to broader Mediterranean maize breeding programs.
- Reduce redundancy; avoid repeating findings already presented in results.
- Link results to biological interpretation (why certain genotypes/traits performed better).
- Reduce overemphasis on obvious statements like “heat reduces yield”.

**Conclusion:**

- Sharpen the take-home message with a clear statement on practical breeding utility.
- Recommend emphasizing key traits (short ASI, high NGP, moderate PH, 1000GW) for future breeding programs.
- Add clear breeding recommendations (e.g., which genotypes/traits are suitable for hybrid development).
- Include broader implication beyond the study location (e.g., similar environments globally).

**Minor Corrections / Specific Improvements-**

Not all are listed below; Please check thoroughly the whole manuscript.

**Abstract**

- “thousandgrain” to “thousand-grain”.
- “representativeness genotype covariates” to “most representative genotype covariates”.
- Break long sentences into shorter sentences for readability.
- Ensure “hydric deficit” term is used consistently.
- Clarify subset of nine Tunisian landraces versus broader collections.
- Ensure tense consistency (past tense for conducted work).
- Correct minor grammar issues: articles, prepositions, plural forms.
- Define abbreviations (e.g., ASI, NGP, PH, GYP) in abstract.

**Introduction**

- “Mediterranean maize landraces are adapted” to “have adapted”.
- Check spelling and consistency of cultivar/landrace names (BK, KAR, MT2, etc.).
- Ensure consistent use of “stress conditions” vs. “stress environments”.

**Materials and Methods**

- Clarify “five checks” -specify whether commercial hybrids or local controls.
- Provide units for all traits measured (e.g., g for biomass, mm for rainfall).
- Ensure consistency in site names (C_22, S_22, D_H, OP) across methods and results.
- Spell out first mention of abbreviations (e.g., DT, DS, GYP, PH, 1000GW, NE, NGP).
- Correct minor grammar and sentence structure issues: “represents” to “represented” where appropriate.

**Results**

- Ensure consistent landrace names throughout (BK, KAR, MT2, GAF, BIZ1, BIZ2, GAB1, GAB2, MT1).
- “showing significant tolerance and adaptability for several stress levels” to “across multiple stress levels”.
- “outperformed for most traits” to “outperformed in most traits”.
- “Other populations, such as GAF, showed a stable grain yield performance” to “Population GAF showed stable grain yield performance”.
- Include units for all traits in tables and figures.
- Check tense consistency: past tense for conducted experiments, present for general knowledge.
- Remove redundancy in statements like “BK maintained highest performance and surpassed checks in yield” (already mentioned).
- Correct minor typographical errors: “cumulated hydric deficit” vs. “cumulative hydric deficit”; “thousand-grain weight” spelled consistently.
- Ensure numbers are consistently formatted (e.g., 1000GW vs. 1,000GW).
- Standardize decimal points (e.g., 1.21 days vs. 1.2 days).
- Make consistent use of % sign with or without a space.

**Discussion**

- Avoid redundant phrasing: “better yields and flowering synchronization” repeated multiple times.
- Shorten excessively long sentences.
- Check all citations for correct format and completeness.
- Correct minor grammatical issues: “plants may adapt by increasing” to “plants appeared to adapt by increasing”.
- Ensure consistent trait naming: “ear number” vs. “number of ears (NE)”, “grain weight” vs. “1000GW”.
- Correct small numeric inconsistencies: e.g., percentages of plant height reduction, ASI, GYP between environments.
- ‘Sumathi et.,2005’ to ‘Sumathi et. al.,2005’
- Replace vague phrases like “significant performance” with specific metrics or values.
- Ensure smooth transition between discussion paragraphs for readability.
- Standardize capitalization in acronyms: e.g., ASI, NGP, PH, GYP, 1000GW throughout the manuscript.
- ‘(Hammami et al., 2025b; Troyer’ complete the reference

**Conclusion**

- Sharpen take-home message; avoid redundancy with discussion.
- Suggest future research directions with precision: e.g., root traits, molecular markers, controlled trials.

**Language & Grammar**

- Proofread entire manuscript for minor grammatical errors: articles, pluralization, verb tense consistency.
- Avoid overly complex or nested sentences; simplify for clarity.
- Check spacing around units, numbers, and symbols.
