# Supplementary material for: Genotypic variability of Tunisian maize landraces: A valuable genetic resource to mitigate drought and heat stress in the Mediterranean basin
Source: PLoS One. 2025 Dec 16;20(12):e0338577. doi: 10.1371/journal.pone.0338577 (PMC12707654; doi:10.1371/journal.pone.0338577)
Supplement: Attachment — Submitted filename: Response_to_Reviewer.docx [file pone.0338577.s008.docx]

**Response to Reviewer Comments**

Dear Reviewer,

We sincerely thank you for your careful reading and constructive suggestions, which have significantly improved the manuscript. Below, we present each of your comments followed by our detailed responses. All suggested changes have been carefully implemented in the revised version of the manuscript.

**Introduction**

**Reviewer:** Clarify the research gap. Link objectives clearly to the breeding relevance under Mediterranean stress conditions.
**Response:** The research gap has been clarified, and the objectives are now explicitly linked to breeding relevance under Mediterranean stress conditions.

**Reviewer:** Clearly state the problem statement (heat stress impact in tropical maize) in the last paragraph.
**Response:** The problem statement regarding the impact of heat stress in tropical maize has been explicitly added in the last paragraph of the Introduction.

**Reviewer:** Include recent references (last 5 years) on heat stress adaptation in maize.
**Response:** Recent references (2019–2024) on heat stress adaptation in maize have been added.

**Reviewer:** Streamline background—reduce long historical references and focus on current breeding challenges.
**Response:** The background section has been streamlined by reducing historical references and focusing on current breeding challenges.

**Materials and Methods**

**Reviewer:** Include exact numbers of plants sampled for each trait and details on checks/controls.
**Response:** The exact number of plants sampled for each trait has been specified, and the type of checks (commercial hybrids and local controls) has been clarified.

**Reviewer:** Specify statistical software/packages used for factorial regression analysis.
**Response:** The statistical software and R packages used for factorial regression analysis have been indicated explicitly.

**Reviewer:** Provide criteria for selecting genotypes (origin, previous evaluation) and explicitly justify why these nine Tunisian landraces were selected.
**Response:** The criteria for selecting the nine Tunisian landraces have been detailed, including their origin and previous evaluations, and the justification for their selection has been added.

**Results**

**Reviewer:** Emphasize patterns and biological interpretations rather than raw percentages.
**Response:** The results have been revised to focus more on biological interpretations and trait patterns rather than raw percentages.

**Reviewer:** Factorial regression interpretation should be illustrated schematically for clarity.
**Response:** A schematic illustration of factorial regression interpretation has been added for clarity.

**Reviewer:** Present key interactions more clearly (focus on PH × DHC, ASI under stress, NE × DAY>40).
**Response:** Key interactions such as PH × DHC, ASI under stress, and NE × DAY>40 have been highlighted more clearly.

**Reviewer:** Remove repetitive mentions of the same superior genotypes in multiple subsections—summarize once.
**Response:** Repetitive mentions of superior genotypes have been removed; they are now summarized once for clarity.

**Reviewer:** Add numeric evidence (means, % changes) when describing genotype performance.
**Response:** Numeric evidence, including means and percentage changes, has been added when describing genotype performance.

**Discussion**

**Reviewer:** Relate observed phenotypic plasticity to physiological mechanisms and breeding implications.
**Response:** The discussion now relates observed phenotypic plasticity to physiological mechanisms and breeding implications.

**Reviewer:** Highlight relevance beyond Tunisia to broader Mediterranean maize breeding programs.
**Response:** The discussion has been extended to highlight the relevance of results beyond Tunisia, particularly for Mediterranean and other stress-prone regions.

**Reviewer:** Reduce redundancy; avoid repeating findings already presented in results.
**Response:** Redundant sentences have been removed to avoid repeating findings already presented in the results.

**Reviewer:** Link results to biological interpretation (why certain genotypes/traits performed better).
**Response:** Results have been linked to biological explanations for the performance of certain genotypes and traits.

**Reviewer:** Reduce overemphasis on obvious statements like “heat reduces yield.”
**Response:** Overly obvious statements were removed or rephrased.

**Conclusion**

**Reviewer:** Sharpen the take-home message with a clear statement on practical breeding utility.
**Response:** The take-home message has been sharpened to emphasize practical breeding utility.

**Reviewer:** Recommend emphasizing key traits (short ASI, high NGP, moderate PH, 1000GW) for future breeding programs.
**Response:** Key traits such as short ASI, high NGP, moderate PH, and 1000GW have been highlighted as priority traits for future breeding programs.

**Reviewer:** Add clear breeding recommendations (e.g., which genotypes/traits are suitable for hybrid development).
**Response:** Breeding recommendations have been added, emphasizing donor lines BK, KAR, and MT2 as suitable for hybrid development.

**Reviewer:** Include broader implication beyond the study location (e.g., similar environments globally).
**Response:** Broader implications were included, extending relevance to Mediterranean drylands and globally stress-prone regions.

**Minor Corrections / Specific Improvements**

**Reviewer:** “thousandgrain” to “thousand-grain”.
**Response:** Corrected.

**Reviewer:** “representativeness genotype covariates” to “most representative genotype covariates.”
**Response:** Corrected.

**Reviewer:** Break long sentences into shorter sentences for readability.
**Response:** Sentences were shortened for better readability.

**Reviewer:** Ensure “hydric deficit” term is used consistently.
**Response:** Standardized throughout the manuscript.

**Reviewer:** Clarify subset of nine Tunisian landraces versus broader collections.
**Response:** Clarified (nine landraces studied from a collection of 223).

**Reviewer:** Ensure tense consistency (past tense for conducted work).
**Response:** Tense was standardized.

**Reviewer:** Correct minor grammar issues: articles, prepositions, plural forms.
**Response:** Corrected.

**Reviewer:** Define abbreviations (e.g., ASI, NGP, PH, GYP) in abstract.
**Response:** Abbreviations have been defined at first mention.

**Reviewer:** “Mediterranean maize landraces are adapted” to “have adapted”.
**Response:** Corrected.

**Reviewer:** Check spelling and consistency of cultivar/landrace names (BK, KAR, MT2, etc.).
**Response:** Corrected throughout.

**Reviewer:** Ensure consistent use of “stress conditions” vs. “stress environments.”
**Response:** Standardized terminology.

**Reviewer:** Clarify “five checks”—specify whether commercial hybrids or local controls.
**Response:** Clarified as a mix of commercial hybrids and local controls.

**Reviewer:** Provide units for all traits measured (e.g., g for biomass, mm for rainfall).
**Response:** Units for all traits were added.

**Reviewer:** Ensure consistency in site names (C_22, S_22, D_H, OP) across methods and results.
**Response:** Corrected and standardized.

**Reviewer:** Spell out first mention of abbreviations (e.g., DT, DS, GYP, PH, 1000GW, NE, NGP).
**Response:** All abbreviations were spelled out at first mention.

**Reviewer:** Correct minor grammar and sentence structure issues: “represents” to “represented” where appropriate.
**Response:** Corrected.

**Reviewer:** Ensure consistent landrace names throughout (BK, KAR, MT2, GAF, BIZ1, BIZ2, GAB1, GAB2, MT1).
**Response:** Corrected throughout.

**Reviewer:** “showing significant tolerance and adaptability for several stress levels” to “across multiple stress levels”.
**Response:** Corrected.

**Reviewer:** “outperformed for most traits” to “outperformed in most traits”.
**Response:** Corrected.

**Reviewer:** “Other populations, such as GAF, showed a stable grain yield performance” to “Population GAF showed stable grain yield performance”.
**Response:** Corrected.

**Reviewer:** Include units for all traits in tables and figures.
**Response:** Units were added consistently.

**Reviewer:** Check tense consistency: past tense for conducted experiments, present for general knowledge.
**Response:** Corrected.

**Reviewer:** Remove redundancy in statements like “BK maintained highest performance and surpassed checks in yield.”
**Response:** Redundancy removed.

**Reviewer:** Correct minor typographical errors: “cumulated hydric deficit” vs. “cumulative hydric deficit”; “thousand-grain weight” consistently.
**Response:** Corrected.

**Reviewer:** Ensure numbers are consistently formatted (e.g., 1000GW vs. 1,000GW).
**Response:** Corrected.

**Reviewer:** Standardize decimal points (e.g., 1.21 days vs. 1.2 days).
**Response:** Standardized.

**Reviewer:** Make consistent use of % sign with or without a space.
**Response:** Corrected consistently.

**Reviewer:** Avoid redundant phrasing: “better yields and flowering synchronization” repeated multiple times.
**Response:** Redundancy removed.

**Reviewer:** Shorten excessively long sentences.
**Response:** Sentences shortened.

**Reviewer:** Check all citations for correct format and completeness.
**Response:** Corrected.

**Reviewer:** Correct minor grammatical issues: “plants may adapt by increasing” to “plants appeared to adapt by increasing.”
**Response:** Corrected.

**Reviewer:** Ensure consistent trait naming: “ear number” vs. “number of ears (NE)”, “grain weight” vs. “1000GW.”
**Response:** Corrected.

**Reviewer:** Correct small numeric inconsistencies: e.g., percentages of plant height reduction, ASI, GYP between environments.
**Response:** Corrected.

**Reviewer:** ‘Sumathi et., 2005’ to ‘Sumathi et al., 2005’.
**Response:** Corrected.

**Reviewer:** Replace vague phrases like “significant performance” with specific metrics or values.
**Response:** Corrected.

**Reviewer:** Ensure smooth transition between discussion paragraphs for readability.
**Response:** Transitions improved.

**Reviewer:** Standardize capitalization in acronyms: e.g., ASI, NGP, PH, GYP, 1000GW throughout the manuscript.
**Response:** Corrected.

**Reviewer:** ‘(Hammami et al., 2025b; Troyer’ complete the reference.
**Response:** Reference completed.

**Reviewer:** Sharpen take-home message; avoid redundancy with discussion.
**Response:** Take-home message sharpened and redundancy avoided.

**Reviewer:** Suggest future research directions with precision: e.g., root traits, molecular markers, controlled trials.
**Response:** Future research directions added with precision.

**Reviewer:** Proofread entire manuscript for minor grammatical errors: articles, pluralization, verb tense consistency.
**Response:** Corrected.

**Reviewer:** Avoid overly complex or nested sentences; simplify for clarity.
**Response:** Sentences simplified.

**Reviewer:** Check spacing around units, numbers, and symbols.
**Response:** Corrected.

In summary, all your concerns, both major and minor, were addressed. The manuscript has been thoroughly revised to improve clarity, scientific accuracy, and breeding relevance.

We are grateful for your valuable feedback, which has greatly contributed to strengthening the manuscript.

Sincerely,

Hammami Mohamed Dhia Eddine
