# Supplementary material for: Genotypic variability of Tunisian maize landraces: A valuable genetic resource to mitigate drought and heat stress in the Mediterranean basin
Source: PLoS One. 2025 Dec 16;20(12):e0338577. doi: 10.1371/journal.pone.0338577 (PMC12707654; doi:10.1371/journal.pone.0338577)
Supplement: Peer review history

# Peer review: Decision Letter 0

**DOI:** 10.1371/journal.pone.0338577.r001

**Authors:** Mehdi Rahimi

24 Jun 2025

Dear Dr. Hammami,

Thank you for submitting your manuscript to PLOS ONE. After careful consideration, we feel that it has merit but does not fully meet PLOS ONE’s publication criteria as it currently stands. Therefore, we invite you to submit a revised version of the manuscript that addresses the points raised during the review process.

Please submit your revised manuscript by Aug 08 2025 11:59PM. If you will need more time than this to complete your revisions, please reply to this message or contact the journal office at plosone@plos.org . When you're ready to submit your revision, log on to https://www.editorialmanager.com/pone/ and select the 'Submissions Needing Revision' folder to locate your manuscript file.

- A rebuttal letter that responds to each point raised by the academic editor and reviewer(s). You should upload this letter as a separate file labeled 'Response to Reviewers'.
- A marked-up copy of your manuscript that highlights changes made to the original version. You should upload this as a separate file labeled 'Revised Manuscript with Track Changes'.
- An unmarked version of your revised paper without tracked changes. You should upload this as a separate file labeled 'Manuscript'.

If you would like to make changes to your financial disclosure, please include your updated statement in your cover letter. Guidelines for resubmitting your figure files are available below the reviewer comments at the end of this letter.

If applicable, we recommend that you deposit your laboratory protocols in protocols.io to enhance the reproducibility of your results. Protocols.io assigns your protocol its own identifier (DOI) so that it can be cited independently in the future. For instructions see: https://journals.plos.org/plosone/s/submission-guidelines#loc-laboratory-protocols . Additionally, PLOS ONE offers an option for publishing peer-reviewed Lab Protocol articles, which describe protocols hosted on protocols.io. Read more information on sharing protocols at https://plos.org/protocols?utm_medium=editorial-email&utm_source=authorletters&utm_campaign=protocols .

We look forward to receiving your revised manuscript.

Kind regards,

Mehdi Rahimi, Ph.D.

Academic Editor

PLOS ONE

Journal Requirements: 

When submitting your revision, we need you to address these additional requirements.

1. Please ensure that your manuscript meets PLOS ONE's style requirements, including those for file naming. The PLOS ONE style templates can be found at

https://journals.plos.org/plosone/s/file?id=wjVg/PLOSOne_formatting_sample_main_body.pdf and

https://journals.plos.org/plosone/s/file?id=ba62/PLOSOne_formatting_sample_title_authors_affiliations.pdf .

2.  We note that the grant information you provided in the ‘Funding Information’ and ‘Financial Disclosure’ sections do not match.

When you resubmit, please ensure that you provide the correct grant numbers for the awards you received for your study in the ‘Funding Information’ section.

3. Thank you for stating the following financial disclosure:

 [This research was funded by MCIU/AEI/ FEDER, UE (PID2022-140991OB-I00) and by PRIMA, a program supported by the European Union under H2020 framework program and PCI2021-121912 funded by MCIN/AEI/ 10.13039/ 501100011033. And by the Agencia Estatal de Investigación (AEI) and the European Fund for Regional Development (FEDER), UE (project code PID2022-140991OB-I00)]. 

Please state what role the funders took in the study.  If the funders had no role, please state: ""The funders had no role in study design, data collection and analysis, decision to publish, or preparation of the manuscript.""

If this statement is not correct you must amend it as needed.

Please include this amended Role of Funder statement in your cover letter; we will change the online submission form on your behalf.

4. In the online submission form, you indicated that [datasets generated during and/or analysed during the current study are available from the corresponding author on reasonable request].

All PLOS journals now require all data underlying the findings described in their manuscript to be freely available to other researchers, either 1. In a public repository, 2. Within the manuscript itself, or 3. Uploaded as supplementary information.

This policy applies to all data except where public deposition would breach compliance with the protocol approved by your research ethics board. If your data cannot be made publicly available for ethical or legal reasons (e.g., public availability would compromise patient privacy), please explain your reasons on resubmission and your exemption request will be escalated for approval.

5. When completing the data availability statement of the submission form, you indicated that you will make your data available on acceptance. We strongly recommend all authors decide on a data sharing plan before acceptance, as the process can be lengthy and hold up publication timelines. Please note that, though access restrictions are acceptable now, your entire data will need to be made freely accessible if your manuscript is accepted for publication. This policy applies to all data except where public deposition would breach compliance with the protocol approved by your research ethics board. If you are unable to adhere to our open data policy, please kindly revise your statement to explain your reasoning and we will seek the editor's input on an exemption. Please be assured that, once you have provided your new statement, the assessment of your exemption will not hold up the peer review process.

6. We notice that your supplementary tables are included in the manuscript file. Please remove them and upload them with the file type 'Supporting Information'. Please ensure that each Supporting Information file has a legend listed in the manuscript after the references list.

[Note: HTML markup is below. Please do not edit.]

Reviewers' comments:

Reviewer's Responses to Questions

**Comments to the Author**

1. Is the manuscript technically sound, and do the data support the conclusions?

Reviewer #1: Partly

**********

2. Has the statistical analysis been performed appropriately and rigorously?

Reviewer #1: No

**********

3. Have the authors made all data underlying the findings in their manuscript fully available?

The PLOS Data policy

Reviewer #1: No

**********

4. Is the manuscript presented in an intelligible fashion and written in standard English?

Reviewer #1: Yes

**********

Reviewer #1: Reviewer’s Report

The manuscript titled "Genotypic variability of Tunisian maize landraces: A Valuable Genetic Resource to Mitigate Drought and Heat Stress in the Mediterranean Basin" addresses a highly relevant and timely topic in the context of climate change and sustainable agriculture. By evaluating the performance of nine Tunisian maize landraces under diverse environmental conditions, including combined drought and heat stress, the study contributes valuable insights into genotype × environment interactions and stress adaptation mechanisms. The use of multi-environment trials, factorial regression, and trait-environment correlation analysis strengthens the scientific rigor and practical relevance of the research. The identification of promising landraces such as BK, KAR, and MT2 adds breeding value to the work, particularly for developing resilient cultivars suited to arid and semi-arid regions. However, several methodological and interpretational limitations need to be addressed to enhance the clarity, depth, and reproducibility of the study.

# Major issues

1. Experimental Design Issues

• Unbalanced Environment Representation:

o Only Mornag had both control and drought stress environments across two years.

o Sousse (optimal) and Gabes (drought + heat) were tested in only one year each introduces unbalanced data and makes statistical modeling more complex.

• Lack of Replication Across Years:

o No repeated testing of optimal and drought+heat environments across multiple years → limits ability to separate year vs. environment effects.

• Single-Year Unique Stress (Gabes):

o Drought + heat environment in Gabes occurred only once; cannot distinguish whether genotype performance is due to stress or year effect.

Clarify this imbalance in the manuscript and you may consider restricting some analyses (e.g., ANOVA, G×E models) to Mornag only, where more balanced replication exists. Use exploratory analyses (e.g., PCA or clustering) for all environments.

Additional concern: Different environments in different years

This adds complexity:

• Year-to-year variation (rainfall, temperature, pests) can mask or mimic stress responses.

• You need to treat year as a factor in your model, but if each environment = one year = one location, you cannot separate these effects.

Unclear total number of entries per block

• With 9 landraces and 5 checks, and 5 blocks:

o Are all 9 landraces planted once across the trial?

• In a typical augmented design, test genotypes (landraces here) are unreplicated, while checks are replicated.

• Need clarity on how 9 landraces were spread over 5 blocks. With 11 rows per block and 5 check rows, only 6 rows are left for test genotypes — but there are 9 landraces in total. This suggests:

o Some blocks might have had 2 landraces, others had 3, or they used more than 5 blocks.

2. Statistical Modeling Limitations

• Use of Augmented Design:

o Augmented design is suitable for unreplicated genotypes but requires appropriate statistical corrections (e.g., adjusted means).

o Block and check structure must be modeled carefully; unclear whether proper error estimation is ensured.

• GGE Biplot Usage:

o While useful, GGE biplot assumes balanced and replicated environments. Using it with unbalanced data (e.g., unique environments like Gabes or Sousse) may bias interpretation.

• No Inclusion of “Year” as a Factor:

o Year should be modeled as a random or fixed effect, especially if the same site is tested over years. Ignoring it inflates error terms and biases G×E estimation.

While the augmented design is appropriate for unreplicated landraces, error control depends heavily on check performance and block correction. Inadequate modeling may inflate error or bias genotype effects. Use adjusted means (e.g., using mixed models or method of Federer) before conducting any further analysis (e.g., PCA, GGE, heatmaps). Use GGE biplot for exploratory insight only. For rigorous G×E analysis, apply tools like AMMI or linear mixed models on the balanced subset (e.g., Mornag). Report this limitation clearly. Model year as a factor in ANOVA or mixed models where applicable. Clearly mention this limitation when interpreting genotype × environment interaction (G×E).

Block size and error estimation may be borderline

• With only 5 blocks and 5 replicated checks:

o Each check is replicated only 5 times,

o This may give a low degrees of freedom (DF) for the error term,

o This affects the reliability of ANOVA, especially if checks are not consistent across blocks.

Acceptable, but limited power for detecting small differences.

Unclear Justification for Heatmap Use

• Use of heatmaps for genotypes and environments is fine visually but can be misleading without statistical backing due to unbalanced data.

Supplement heatmaps with clustering or PCA for robustness.

3. Analytical Scope Limitations

• Mixing Different Stress Types:

o Combining single-year optimal/drought+heat with multi-year drought trials may obscure true G×E signals and reduce model power.

Avoid over-interpreting results from single-season locations. Use seasonal replication (Mornag) for robust inference; present other data as supplemental.

# Other important issues (not major but needed to be addressed properly)

Abstract

• Add experimental design info in abstract. No info on replications, plot size, or statistical rigor besides factorial regression. Briefly mention design type (e.g., RCBD with replications) to support validity.

• Line 37: Drought and heat stress…. This line should go later

• Keep consistency in estimates/naming i.e., hydric deficit and water deficit

• No yield ranges, ASI values, or grain weight metrics are included. Include a few key metrics to quantify performance differences (e.g., % yield reduction under stress).

• “Drought and heat stress” are combined in analysis without clear distinction. Clarify whether these stresses occurred independently or in combination and how that was addressed analytically.

• The term “possible stress tolerance mechanisms” is used, but no mechanism is clearly defined. Mention at least one inferred mechanism (e.g., shorter ASI, high TGW under stress).

• It’s not immediately clear whether these are traditional landraces, improved cultivars, or selections. Specify the genetic background to highlight their breeding relevance.

• GAF is mentioned for stable performance but lacks interpretation. Indicate why stable performance under moderate stress is valuable (e.g., for marginal zones).

M&M

• Line 106-107: “Each block consisted of 11 rows and included five genotypes per row.”

• Line 122-123: you mentioned frequent heat waves for the Gabes location but the temperature around 35 C. Need explanation

• Mean data of environmental covariates for different locations were not present in the manuscript which is needed.

• Analyzed 10 traits, out of which 5 traits were presented in details environment-wise and others were mean over six environments. Any specific reason? Need clarification. in details environment-wise data required to interpret the variations across the environments specially traits like ASI.

• Line 196: correct the citation style

• Line 196 and line 210: same software RStudio but different citation number

• Spacing Assumption:

o Claimed plant density of 60,000 plants/ha is only valid if thinning is done precisely.

o Spacing of 70 cm × 25 cm with 12 hills/row matches this density, but depends on row length and number — needs explicit validation.

Provide detailed field layout and validate plant density calculations. Include actual stand counts if possible.

Data Integrity / Interpretation Concerns:

• Rainfall Variation Doubts:

o You noted rainfall differences were not substantial, yet the environment was classified as “drought” this might be misleading unless supported by soil moisture or crop stress data.

• Control vs. Optimal Definition Ambiguity:

o The distinction between “control” and “optimal” seems unclear if climatic conditions were similar — might raise reviewer concerns about justification for separate environmental categories.

Justify environment classification. Alternatively, consider merging or reclassifying environments based on similarity.

Results

• Although the ANOVA shows significant G×E interactions for some traits (DT, DS, PH, 1000GW, GYP), the strength and practical implications of these interactions are not quantified or deeply interpreted. Include interaction plots or variance component breakdowns to illustrate the contribution and pattern of G×E effects more clearly.

• Heatmap interpretations are largely qualitative and based on color intensity, which may be subjective. Supplement the heatmap findings with numerical cluster validation methods (e.g., silhouette scores or hierarchical clustering dendrograms) to substantiate clustering.

• Genotypic stability is inferred from proximity to the origin in GGE biplots without using a defined stability index. Calculate and report numerical stability parameters (e.g., Shukla’s stability variance or AMMI stability index) to confirm visual assessments.

• The clusters (A, B, C) identified in the heatmap are not quantitatively validated or connected to broader genetic groupings (e.g., landrace origin, maturity group). Conduct a PCA or clustering analysis based on both phenotypic and environmental responses to support the classification.

• Genotype performance is described visually, but no tables with numerical rankings or genotype × environment scores are provided. Add a supplementary table showing PC1 and PC2 scores or genotype rankings across environments for transparency.

• While factorial regression results are discussed, there's no R², AIC, BIC, or other model fitness indicators reported. Include model fit statistics and possibly residual plots to validate the regression model.

• The results briefly mention that some traits had no significant ENV × GEN effects but don’t report which traits or data. Clarify which traits were not significantly affected by ENV × GEN and provide their data (possibly in a supplementary table).

• Some data are missing in the mean table. Reason behind it and how it was handled during the analysis.

Discussion

• The text is dense and often uses long, complex sentences that may reduce readability. Simplify sentence structures and break down long paragraphs for better clarity and reader engagement.

• Significant interaction terms (e.g., PH × DHC, NE × DAY>40) are reported but not biologically contextualized. Briefly explain the biological implications of these interactions (e.g., why PH × DHC has a negative effect) in the results or discussion.

• Factorial regression and GGE biplot results are mentioned, but no statistics (e.g., F-values, R², significance levels) are discussed. Include key statistical metrics to substantiate claims and support interpretations.

• Previous studies citations like [23], [37], [39], etc., and others are used frequently but are not always well integrated into the discussion. Briefly explain the relevance of each cited study and how it supports or contrasts with the findings.

• The discussion spans multiple traits and interactions without summarizing the most impactful results. Add a concluding paragraph that succinctly recaps the top-performing genotypes and most significant trait–environment interactions.

• While the associations/connections between traits and yield are described, the underlying physiological or molecular mechanisms are not discussed. Provide hypotheses or references to explain mechanisms (e.g., why shorter plants perform better under stress).

• Although high-performing landraces are identified, the discussion lacks clear breeding strategy recommendations. Include concrete implications for future breeding efforts, such as which traits to prioritize and potential cross combinations.

• Some traits like ASI and 1000GW are discussed in depth, while others like NGP or AB get little attention. Ensure all analyzed traits receive proportionate discussion, especially those contributing significantly to yield variability.

• Statements about genetic similarity to reference populations (e.g., Reid Yellow Dent) are broad. Include metrics or PCA/phylogenetic figures to substantiate claims of genetic similarity and diversity.

• The ending paragraph is abrupt and doesn’t effectively summarize or transition to conclusions. Add a transition to the conclusion emphasizing the relevance of findings to climate-resilient maize production.

Conclusion

• The conclusion makes general claims (e.g., “better yields,” “significant tolerance”) without citing quantitative results or specific statistical outcomes. Include one or two key figures or percentage improvements in yield or ASI to strengthen the claims.

• It mentions “main effect of drought stress” as a limitation but does not clearly distinguish between heat and drought or how their interaction was handled. Clarify whether factorial designs or separate stress-only conditions were used and recommend controlled drought-only trials for future work.

• The only limitation mentioned is the drought-heat overlap, with no reference to possible experimental design constraints, sample size, or genotypic variability limitations. Add 1–2 more concrete limitations—e.g., lack of molecular validation, absence of root traits or physiology-based assessments, or short-term field evaluation—and how future work could address them.

• The conclusion touches briefly on practical applications but lacks depth in addressing policy, breeding program integration, or climate resilience strategies. Expand with a statement like: “These findings can guide national maize breeding programs aiming to develop climate-resilient varieties tailored for North African drylands.”

• Phrases such as “drought tolerance,” “adaptability to stressed conditions,” and “resilience to climatic changes” are repeated without variation or deeper insight. Rephrase and diversify wording. For example:

o “...resilient performance under compound abiotic stresses...”

o “...robust reproductive synchronization despite thermal and hydric challenges...”

• While the conclusion mentions potential for breeding, it doesn’t outline next steps or specific breeding strategies. Add a forward-looking statement like:

“Future work should focus on integrating these landraces into pre-breeding pipelines, leveraging marker-assisted selection or genomic prediction tools to accelerate drought-resilience trait introgression.”

• The final sentence is too generic (“showing more adaptability to stressed conditions”), lacking a strong, memorable message. Strengthen the closing line to reflect a clear takeaway:

“Overall, this study lays the groundwork for leveraging native Tunisian maize diversity to safeguard future maize production under increasing climatic pressures.”

References

Some inconsistencies were observed in the bibliography formatting. Please be in line with the journals specific format. Double check the reference section.

Tables and figures

Table numbers started with “S” which generally specifies for supplementary tables. Need correction.

Figures quality was not up to the mark. Must be improved

**********

what does this mean? ). If published, this will include your full peer review and any attached files.

If you choose “no”, your identity will remain anonymous but your review may still be made public.

**Do you want your identity to be public for this peer review?** For information about this choice, including consent withdrawal, please see our Privacy Policy

Reviewer #1: **Yes: ** Md. Ashraful Alam

**********

[NOTE: If reviewer comments were submitted as an attachment file, they will be attached to this email and accessible via the submission site. Please log into your account, locate the manuscript record, and check for the action link "View Attachments". If this link does not appear, there are no attachment files.]

While revising your submission, please upload your figure files to the Preflight Analysis and Conversion Engine (PACE) digital diagnostic tool, https://pacev2.apexcovantage.com/ . PACE helps ensure that figures meet PLOS requirements. To use PACE, you must first register as a user. Registration is free. Then, login and navigate to the UPLOAD tab, where you will find detailed instructions on how to use the tool. If you encounter any issues or have any questions when using PACE, please email PLOS at figures@plos.org . Please note that Supporting Information files do not need this step.

Attachment Submitted filename: Reviewers report.docx

# Author comment: Author response to Decision Letter 1

**DOI:** 10.1371/journal.pone.0338577.r002

13 Aug 2025

Response Letter to Reviewer

Reviewer Comment:

The manuscript titled "Genotypic variability of Tunisian maize landraces: A Valuable Genetic Resource to Mitigate Drought and Heat Stress in the Mediterranean Basin" addresses a highly relevant and timely topic in the context of climate change and sustainable agriculture. By evaluating the performance of nine Tunisian maize landraces under diverse environmental conditions, including combined drought and heat stress, the study contributes valuable insights into genotype × environment interactions and stress adaptation mechanisms. The use of multi-environment trials, factorial regression, and trait-environment correlation analysis strengthens the scientific rigor and practical relevance of the research. The identification of promising landraces such as BK, KAR, and MT2 adds breeding value to the work, particularly for developing resilient cultivars suited to arid and semi-arid regions. However, several methodological and interpretational limitations need to be addressed to enhance the clarity, depth, and reproducibility of the study.

# Major issues

1. Experimental Design Issues

• Unbalanced Environment Representation:

o Only Mornag had both control and drought stress environments across two years.

o Sousse (optimal) and Gabes (drought + heat) were tested in only one year each introduces unbalanced data and makes statistical modeling more complex.

• Lack of Replication Across Years:

o No repeated testing of optimal and drought+heat environments across multiple years → limits ability to separate year vs. environment effects.

• Single-Year Unique Stress (Gabes):

o Drought + heat environment in Gabes occurred only once; cannot distinguish whether genotype performance is due to stress or year effect.

Clarify this imbalance in the manuscript and you may consider restricting some analyses (e.g., ANOVA, G×E models) to Mornag only, where more balanced replication exists. Use exploratory analyses (e.g., PCA or clustering) for all environments.

Response to Reviewer:

We appreciate the reviewer’s observations regarding the unbalanced representation of environments and the lack of replication across years. However, we would like to clarify that the main objective of our study is not to compare specific geographic locations, but rather to evaluate the performance of Mediterranean maize landraces under distinct cropping conditions, which were intentionally created or naturally occurred in each site.

More specifically:

In Mornag, the experiment was conducted in both 2022 and 2023 under two water regimes: moderate water stress with coinciding heat waves (S_22 and S_23), and full irrigation with heat waves (C_22 and C_23). This allowed us to evaluate genotype performance under reproducible, yet contrasting, stress conditions.

• The optimal environment (Sousse, 2022) and the combined drought and heat stress (Gabes, 2022) were included to broaden the range of Mediterranean growing conditions, even if they were tested in only one year. These environments represent important abiotic stress scenarios in Mediterranean agriculture.

Therefore, the intention was not to analyze regional or year effects, but rather to understand genotypic responses across six distinct Mediterranean cropping conditions, which we treated as fixed environments in the statistical analysis. This aligns with the goal of identifying landraces with superior performance under specific abiotic stress combinations.

To clarify this in the manuscript, we have added Table 4: Agro-Climatic Description and Abiotic Stress Levels of Experimental Environments, which provides a detailed characterization of each environment and justifies their inclusion in the study.

Reviewer Comment:

Additional concern: Different environments in different years

This adds complexity:

• Year-to-year variation (rainfall, temperature, pests) can mask or mimic stress responses.

• You need to treat year as a factor in your model, but if each environment = one year = one location, you cannot separate these effects.

Response to Reviewer:

We appreciate the reviewer’s concern regarding the potential confounding between year, location, and environment. However, we would like to clarify that in this study, our aim was not to analyze year effects, but rather to create and capture a wider range of Mediterranean cropping conditions, including various combinations of water regimes and thermal stress.

The two additional environments in 2023 (C_23 and S_23) were not introduced to study year-to-year variation, but rather to increase environmental diversity and enrich the evaluation of landrace performance under relevant stress scenarios. Therefore, we do not consider it necessary to include “year” as a factor in the model, since the environments themselves defined by their agro-climatic characteristics and stress levels are the primary focus of our analysis.

The observed imbalance in environmental representation is due to the fact that the Mornag locality was the only site where we were able to conduct trials under both control and drought conditions across two consecutive years. We considered it valuable to have at least one location with replicated trials under both stress and non-stress conditions, in order to strengthen the reliability of comparisons in these two key environments. Moreover, in our model, environments were treated as fixed factors, and our goal was to compare and identify landraces that perform well under six distinct environmental scenarios, rather than to study temporal effects. This has been clarified in the revised version of the manuscript, along with the inclusion of Table 4, which provides a detailed agro-climatic description of all environments to support this interpretation.

Reviewer Comment:

Unclear total number of entries per block

• With 9 landraces and 5 checks, and 5 blocks:

o Are all 9 landraces planted once across the trial?

• In a typical augmented design, test genotypes (landraces here) are unreplicated, while checks are replicated.

• Need clarity on how 9 landraces were spread over 5 blocks. With 11 rows per block and 5 check rows, only 6 rows are left for test genotypes — but there are 9 landraces in total. This suggests:

o Some blocks might have had 2 landraces, others had 3, or they used more than 5 blocks.

Response to Reviewer:

We thank the reviewer for pointing out the need for greater clarity regarding the experimental design.

We would like to clarify that the Tunisian landraces analyzed in this study represent a subset (9 entries) of a larger panel of 255 Mediterranean varieties and selections cycles evaluated in the full trial. For the purpose of this manuscript, the analysis focused specifically on the 9 Tunisian landraces, as the objective was to assess their performance under different Mediterranean stress conditions.

The trial was conducted using an augmented design over two consecutive years. In the first year, the design included 5 blocks with 54 plots per block. Of these, 3 plots in each block were allocated to check varieties, which were replicated across all blocks, while the remaining 51 plots were assigned to unreplicated landraces. In the second year, the augmented design consisted of 55 plots per block, with 4 plots assigned to replicated check varieties and 51 plots allocated to unreplicated landraces. The remaining plots in both years were used to randomly assign the landraces across blocks.

Since the 9 Tunisian landraces were not replicated, their distribution varied slightly across environments. For example, in environment C_22, the distribution was as follows:

Block 1: BIZ1, BK

Block 2: GAB2, GAF

Block 3: KAR, GAB1

Block 4: BIZ2, MT1

Block 5: MT2

In other environments, a different random assignment of landraces was used, always respecting the augmented design structure.

We have revised and clarified the Field Trials section in the manuscript to better reflect this structure and improve transparency for the reader.

Reviewer Comment:

2. Statistical Modeling Limitations

• Use of Augmented Design:

o Augmented design is suitable for unreplicated genotypes but requires appropriate statistical corrections (e.g., adjusted means).

o Block and check structure must be modeled carefully; unclear whether proper error estimation is ensured.

Response to Reviewer:

We appreciate the reviewer’s comment regarding the use of the augmented design. In our study, this design was chosen due to the large number of landraces evaluated, which made full replication logistically unfeasible. To ensure reliable error estimation and accurate adjustment of means, we included replicated checks in each of the five blocks. These checks enabled the estimation of block effects and error variance, which were used to adjust the means of the unreplicated landraces. Least Squares Means (LS means) were calculated using the Proc Mixed procedure in SAS, treating genotypes and environments as fixed effects while blocks nested within environments as Random effect. To compare the LS means of the landraces we calculate the Least Significant Difference (LSD) at P < 0.05 using the checks. Furthermore, for traits showing a significant genotype × environment interaction, separate analyses of variance were conducted within each environment while for traits without significant interaction, the LSD was derived from the combined analysis across environments.

Reviewer Comment:

• GGE Biplot Usage:

o While useful, GGE biplot assumes balanced and replicated environments. Using it with unbalanced data (e.g., unique environments like Gabes or Sousse) may bias interpretation.

Response to Reviewer:

We thank the reviewer for this important comment. In our study, GGE biplot analysis was used to evaluate genotype × environment interactions, focusing on both the "Mean vs. Stability" and "Which-won-where" views to identify superior landraces under varying conditions. Our objective was not to compare specific locations, but rather to assess genotypic performance across distinct agronomic conditions that represent contrasting Mediterranean stress scenarios. To address the unbalanced nature of the data, LS means (Least Squares Means) adjusted per block were used as input for the GGE biplot analysis, ensuring a more accurate comparison despite the lack of full replication.

Reviewer Comment:

• No Inclusion of “Year” as a Factor:

o Year should be modeled as a random or fixed effect, especially if the same site is tested over years. Ignoring it inflates error terms and biases G×E estimation.

Response to Reviewer:

We thank the reviewer for highlighting this important point. The primary objective of our study was to evaluate the performance and stability of Mediterranean maize landraces under contrasting agro-environmental conditions.

While the effect of “year” was not modeled separately, each environment (C_22, C_23, S_22, S_23, OP, D_H) was deliberately defined as a unique combination of year × management conditions, representing distinct Mediterranean stress scenarios (such as moderate drought, combined drought and heat, or optimal conditions). Therefore, we treated “environment” as a fixed effect characterizing these specific growing conditions, rather than as a proxy for year or site. The repetition of the trial in 2023 at the same location (Mornag) under both full irrigation and moderate drought (C_23 and S_23) was intended to expand the range of environments, not to assess year effects per se. This design choice allowed us to increase environmental diversity and strengthen genotype evaluation under relevant Mediterranean scenarios.

We acknowledge that this approach does not allow a complete separation of year and environment effects. However, we believe this limitation does not undermine the main objective of the study, which was to compare genotype performance and stability across well-characterized and representative stress environments.

Reviewer Comment:

While the augmented design is appropriate for unreplicated landraces, error control depends heavily on check performance and block correction. Inadequate modeling may inflate error or bias genotype effects. Use adjusted means (e.g., using mixed models or method of Federer) before conducting any further analysis (e.g., PCA, GGE, heatmaps). Use GGE biplot for exploratory insight only. For rigorous G×E analysis, apply tools like AMMI or linear mixed models on the balanced subset (e.g., Mornag). Report this limitation clearly. Model year as a factor in ANOVA or mixed models where applicable. Clearly mention this limitation when interpreting genotype × environment interaction (G×E).

Block size and error estimation may be borderline

• With only 5 blocks and 5 replicated checks:

o Each check is replicated only 5 times,

o This may give a low degrees of freedom (DF) for the error term,

o This affects the reliability of ANOVA, especially if checks are not consistent across blocks.

Acceptable, but limited power for detecting small differences.

Unclear Justification for Heatmap Use

• Use of heatmaps for genotypes and environments is fine visually but can be misleading without statistical backing due to unbalanced data.

Supplement heatmaps with clustering or PCA for robustness.

3. Analytical Scope Limitations

• Mixing Different Stress Types:

o Combining single-year optimal/drought+heat with multi-year drought trials may obscure true G×E signals and reduce model power.

Avoid over-interpreting results from single-season locations. Use seasonal replication (Mornag) for robust inference; present other data as supplemental.

Response to Reviewer:

We thank the reviewer for the detailed and constructive comments regarding the augmented design and subsequent analyses.

As outlined in the revised Materials and Methods section, the experiment was conducted using an augmented design with five blocks. Within each block, 3 plots (in 2022) or 4 plots (in 2023) were allocated to check varieties, which were replicated across all five blocks, ensuring 15 and 20 replicated plots per environment. The remaining plots in each block were used to randomly assign the unreplicated landraces. This design allowed estimation of block effects and error variance using the replicated checks, enabling adjustment of landrace means and valid within-environment comparisons. The Least Squares Means (LS means) were obtained using the Proc Mixed procedure in SAS, treating blocks nested within environments as Random effect and environments and genotypes as fixed effects.

Regarding G×E interaction, we used GGE biplot analysis as an exploratory tool to visualize genotype performance and stability across environments. These environments were not treated merely as years or sites, but as unique combinations of year × water/heat regime (e.g., C_22, S_23, OP, D_H), reflecting distinct Mediterranean stress scenarios. The LS means adjusted per blocks were used as input in the GGE biplot, and we clarify that interpretations are exploratory and should be viewed with this limitation in mind.

Concerning the heatmaps, we agree that unbalanced data can lead to misleading visual interpretations if not appropriately treated. To reinforce the analysis, we conducted a hierarchical clustering of genotypes based on their adjusted LS means adjusted per blocks across environments. These clusters were integrated into the heatmap visualization and are now included in the Supplementary Material (S1 Fig) to support the robustness of the visual analysis.

Lastly, we acknowledge that year effects were not modeled as a separate factor. Instead, "environments" in our study were defined as specific combinations of year × treatment, and were treated as fixed.

Reviewer Comment:

# Other important issues (not major but needed to be addressed properly)

Abstract

• Add experimental design info in abstract. No info on replications, plot size, or statistical rigor besides factorial regression. Briefly mention design type (e.g., RCBD with replications) to support validity.

• Line 37: Drought and heat stress…. This line should go later

• Keep consistency in estimates/naming i.e., hydric deficit and water deficit

• No yield ranges, ASI values, or grain weight metrics are included. Include a few key metrics to quantify performance differences (

Attachment Submitted filename: Response Letter to Reviewers.docx

# Peer review: Decision Letter 1

**DOI:** 10.1371/journal.pone.0338577.r003

**Authors:** Mehdi Rahimi

7 Sep 2025

Dear Dr. Hammami,

Please submit your revised manuscript by Oct 22 2025 11:59PM. If you will need more time than this to complete your revisions, please reply to this message or contact the journal office at plosone@plos.org . When you're ready to submit your revision, log on to https://www.editorialmanager.com/pone/ and select the 'Submissions Needing Revision' folder to locate your manuscript file.

- A rebuttal letter that responds to each point raised by the academic editor and reviewer(s). You should upload this letter as a separate file labeled 'Response to Reviewers'.
- A marked-up copy of your manuscript that highlights changes made to the original version. You should upload this as a separate file labeled 'Revised Manuscript with Track Changes'.
- An unmarked version of your revised paper without tracked changes. You should upload this as a separate file labeled 'Manuscript'.

If applicable, we recommend that you deposit your laboratory protocols in protocols.io to enhance the reproducibility of your results. Protocols.io assigns your protocol its own identifier (DOI) so that it can be cited independently in the future. For instructions see: https://journals.plos.org/plosone/s/submission-guidelines#loc-laboratory-protocols . Additionally, PLOS ONE offers an option for publishing peer-reviewed Lab Protocol articles, which describe protocols hosted on protocols.io. Read more information on sharing protocols at https://plos.org/protocols?utm_medium=editorial-email&utm_source=authorletters&utm_campaign=protocols .

We look forward to receiving your revised manuscript.

Kind regards,

Mehdi Rahimi, Ph.D.

Academic Editor

PLOS ONE

Journal Requirements:

If the reviewer comments include a recommendation to cite specific previously published works, please review and evaluate these publications to determine whether they are relevant and should be cited. There is no requirement to cite these works unless the editor has indicated otherwise. 

Please review your reference list to ensure that it is complete and correct. If you have cited papers that have been retracted, please include the rationale for doing so in the manuscript text, or remove these references and replace them with relevant current references. Any changes to the reference list should be mentioned in the rebuttal letter that accompanies your revised manuscript. If you need to cite a retracted article, indicate the article’s retracted status in the References list and also include a citation and full reference for the retraction notice.

[Note: HTML markup is below. Please do not edit.]

Reviewers' comments:

Reviewer's Responses to Questions

**Comments to the Author**

Reviewer #1: All comments have been addressed

**********

2. Is the manuscript technically sound, and do the data support the conclusions?

Reviewer #1: Partly

**********

3. Has the statistical analysis been performed appropriately and rigorously?

Reviewer #1: I Don't Know

**********

4. Have the authors made all data underlying the findings in their manuscript fully available?

The PLOS Data policy

Reviewer #1: Yes

**********

5. Is the manuscript presented in an intelligible fashion and written in standard English?

Reviewer #1: Yes

**********

Reviewer #1: Thank you for submitting the revised manuscript. The revisions have substantially improved clarity, particularly in the results and discussion sections, with better explanation of trait performance under stress. The abstract, tables, and figures are now clearer and more consistent, and key were well-highlighted. A few minor corrections regarding terminology consistency, grammar issues, typo etc., which will further polish the manuscript. Below is my concern which needed to be addressed.

Major Concern-

Introduction:

• Clarify the research gap Link objectives clearly to the breeding relevance under Mediterranean stress conditions.

• Clearly state the problem statement (heat stress impact in tropical maize) in the last paragraph.

• Include recent references (last 5 years) on heat stress adaptation in maize.

• Streamline background- reduce long historical references and focus on current breeding challenges.

Materials and Methods:

• Include exact numbers of plants sampled for each trait and details on checks/controls.

• Specify statistical software/packages used for factorial regression analysis.

• Provide criteria for selecting genotypes (origin, previous evaluation) and explicitly justify why these nine Tunisian landraces were selected.

Results:

• Emphasize patterns and biological interpretations rather than raw percentages;

• Factorial regression interpretation should be illustrated schematically for clarity.

• Present key interactions more clearly (focus on PH × DHC, ASI under stress, NE × DAY>40).

• Remove repetitive mentions of the same superior genotypes in multiple subsections-summarize once.

• Add numeric evidence (means, % changes) when describing genotype performance.

Discussion:

• Relate observed phenotypic plasticity to physiological mechanisms and breeding implications.

• Highlight relevance beyond Tunisia to broader Mediterranean maize breeding programs.

• Reduce redundancy; avoid repeating findings already presented in results.

• Link results to biological interpretation (why certain genotypes/traits performed better).

• Reduce overemphasis on obvious statements like “heat reduces yield”.

Conclusion:

• Sharpen the take-home message with a clear statement on practical breeding utility.

• Recommend emphasizing key traits (short ASI, high NGP, moderate PH, 1000GW) for future breeding programs.

• Add clear breeding recommendations (e.g., which genotypes/traits are suitable for hybrid development).

• Include broader implication beyond the study location (e.g., similar environments globally).

Minor Corrections / Specific Improvements-

Not all are listed below; Please check thoroughly the whole manuscript.

Abstract

• “thousandgrain” to “thousand-grain”.

• “representativeness genotype covariates” to “most representative genotype covariates”.

• Break long sentences into shorter sentences for readability.

• Ensure “hydric deficit” term is used consistently.

• Clarify subset of nine Tunisian landraces versus broader collections.

• Ensure tense consistency (past tense for conducted work).

• Correct minor grammar issues: articles, prepositions, plural forms.

• Define abbreviations (e.g., ASI, NGP, PH, GYP) in abstract.

Introduction

• “Mediterranean maize landraces are adapted” to “have adapted”.

• Check spelling and consistency of cultivar/landrace names (BK, KAR, MT2, etc.).

• Ensure consistent use of “stress conditions” vs. “stress environments”.

Materials and Methods

• Clarify “five checks” -specify whether commercial hybrids or local controls.

• Provide units for all traits measured (e.g., g for biomass, mm for rainfall).

• Ensure consistency in site names (C_22, S_22, D_H, OP) across methods and results.

• Spell out first mention of abbreviations (e.g., DT, DS, GYP, PH, 1000GW, NE, NGP).

• Correct minor grammar and sentence structure issues: “represents” to “represented” where appropriate.

Results

• Ensure consistent landrace names throughout (BK, KAR, MT2, GAF, BIZ1, BIZ2, GAB1, GAB2, MT1).

• “showing significant tolerance and adaptability for several stress levels” to “across multiple stress levels”.

• “outperformed for most traits” to “outperformed in most traits”.

• “Other populations, such as GAF, showed a stable grain yield performance” to “Population GAF showed stable grain yield performance”.

• Include units for all traits in tables and figures.

• Check tense consistency: past tense for conducted experiments, present for general knowledge.

• Remove redundancy in statements like “BK maintained highest performance and surpassed checks in yield” (already mentioned).

• Correct minor typographical errors: “cumulated hydric deficit” vs. “cumulative hydric deficit”; “thousand-grain weight” spelled consistently.

• Ensure numbers are consistently formatted (e.g., 1000GW vs. 1,000GW).

• Standardize decimal points (e.g., 1.21 days vs. 1.2 days).

• Make consistent use of % sign with or without a space.

Discussion

• Avoid redundant phrasing: “better yields and flowering synchronization” repeated multiple times.

• Shorten excessively long sentences.

• Check all citations for correct format and completeness.

• Correct minor grammatical issues: “plants may adapt by increasing” to “plants appeared to adapt by increasing”.

• Ensure consistent trait naming: “ear number” vs. “number of ears (NE)”, “grain weight” vs. “1000GW”.

• Correct small numeric inconsistencies: e.g., percentages of plant height reduction, ASI, GYP between environments.

• ‘Sumathi et.,2005’ to ‘Sumathi et. al.,2005’

• Replace vague phrases like “significant performance” with specific metrics or values.

• Ensure smooth transition between discussion paragraphs for readability.

• Standardize capitalization in acronyms: e.g., ASI, NGP, PH, GYP, 1000GW throughout the manuscript.

• ‘(Hammami et al., 2025b; Troyer’ complete the reference

Conclusion

• Sharpen take-home message; avoid redundancy with discussion.

• Suggest future research directions with precision: e.g., root traits, molecular markers, controlled trials.

Language & Grammar

• Proofread entire manuscript for minor grammatical errors: articles, pluralization, verb tense consistency.

• Avoid overly complex or nested sentences; simplify for clarity.

• Check spacing around units, numbers, and symbols.

**********

what does this mean? ). If published, this will include your full peer review and any attached files.

If you choose “no”, your identity will remain anonymous but your review may still be made public.

**Do you want your identity to be public for this peer review?** For information about this choice, including consent withdrawal, please see our Privacy Policy

Reviewer #1: **Yes: ** Md. Ashraful Alam

**********

[NOTE: If reviewer comments were submitted as an attachment file, they will be attached to this email and accessible via the submission site. Please log into your account, locate the manuscript record, and check for the action link "View Attachments". If this link does not appear, there are no attachment files.]

While revising your submission, please upload your figure files to the Preflight Analysis and Conversion Engine (PACE) digital diagnostic tool, https://pacev2.apexcovantage.com/ . PACE helps ensure that figures meet PLOS requirements. To use PACE, you must first register as a user. Registration is free. Then, login and navigate to the UPLOAD tab, where you will find detailed instructions on how to use the tool. If you encounter any issues or have any questions when using PACE, please email PLOS at figures@plos.org

Attachment Submitted filename: Reviewer comments_26.8.25.docx

# Author comment: Author response to Decision Letter 2

**DOI:** 10.1371/journal.pone.0338577.r004

5 Nov 2025

Dear Reviewer,

We sincerely thank you for your careful reading and constructive suggestions, which have significantly improved the manuscript. Below, we present each of your comments followed by our detailed responses. All suggested changes have been carefully implemented in the revised version of the manuscript.

Introduction

Reviewer: Clarify the research gap. Link objectives clearly to the breeding relevance under Mediterranean stress conditions.

Response: The research gap has been clarified, and the objectives are now explicitly linked to breeding relevance under Mediterranean stress conditions.

Reviewer: Clearly state the problem statement (heat stress impact in tropical maize) in the last paragraph.

Response: The problem statement regarding the impact of heat stress in tropical maize has been explicitly added in the last paragraph of the Introduction.

Reviewer: Include recent references (last 5 years) on heat stress adaptation in maize.

Response: Recent references (2019–2024) on heat stress adaptation in maize have been added.

Reviewer: Streamline background—reduce long historical references and focus on current breeding challenges.

Response: The background section has been streamlined by reducing historical references and focusing on current breeding challenges.

Materials and Methods

Reviewer: Include exact numbers of plants sampled for each trait and details on checks/controls.

Response: The exact number of plants sampled for each trait has been specified, and the type of checks (commercial hybrids and local controls) has been clarified.

Reviewer: Specify statistical software/packages used for factorial regression analysis.

Response: The statistical software and R packages used for factorial regression analysis have been indicated explicitly.

Reviewer: Provide criteria for selecting genotypes (origin, previous evaluation) and explicitly justify why these nine Tunisian landraces were selected.

Response: The criteria for selecting the nine Tunisian landraces have been detailed, including their origin and previous evaluations, and the justification for their selection has been added.

Results

Reviewer: Emphasize patterns and biological interpretations rather than raw percentages.

Response: The results have been revised to focus more on biological interpretations and trait patterns rather than raw percentages.

Reviewer: Factorial regression interpretation should be illustrated schematically for clarity.

Response: A schematic illustration of factorial regression interpretation has been added for clarity.

Reviewer: Present key interactions more clearly (focus on PH × DHC, ASI under stress, NE × DAY>40).

Response: Key interactions such as PH × DHC, ASI under stress, and NE × DAY>40 have been highlighted more clearly.

Reviewer: Remove repetitive mentions of the same superior genotypes in multiple subsections—summarize once.

Response: Repetitive mentions of superior genotypes have been removed; they are now summarized once for clarity.

Reviewer: Add numeric evidence (means, % changes) when describing genotype performance.

Response: Numeric evidence, including means and percentage changes, has been added when describing genotype performance.

Discussion

Reviewer: Relate observed phenotypic plasticity to physiological mechanisms and breeding implications.

Response: The discussion now relates observed phenotypic plasticity to physiological mechanisms and breeding implications.

Reviewer: Highlight relevance beyond Tunisia to broader Mediterranean maize breeding programs.

Response: The discussion has been extended to highlight the relevance of results beyond Tunisia, particularly for Mediterranean and other stress-prone regions.

Reviewer: Reduce redundancy; avoid repeating findings already presented in results.

Response: Redundant sentences have been removed to avoid repeating findings already presented in the results.

Reviewer: Link results to biological interpretation (why certain genotypes/traits performed better).

Response: Results have been linked to biological explanations for the performance of certain genotypes and traits.

Reviewer: Reduce overemphasis on obvious statements like “heat reduces yield.”

Response: Overly obvious statements were removed or rephrased.

Conclusion

Reviewer: Sharpen the take-home message with a clear statement on practical breeding utility.

Response: The take-home message has been sharpened to emphasize practical breeding utility.

Reviewer: Recommend emphasizing key traits (short ASI, high NGP, moderate PH, 1000GW) for future breeding programs.

Response: Key traits such as short ASI, high NGP, moderate PH, and 1000GW have been highlighted as priority traits for future breeding programs.

Reviewer: Add clear breeding recommendations (e.g., which genotypes/traits are suitable for hybrid development).

Response: Breeding recommendations have been added, emphasizing donor lines BK, KAR, and MT2 as suitable for hybrid development.

Reviewer: Include broader implication beyond the study location (e.g., similar environments globally).

Response: Broader implications were included, extending relevance to Mediterranean drylands and globally stress-prone regions.

Minor Corrections / Specific Improvements

Reviewer: “thousandgrain” to “thousand-grain”.

Response: Corrected.

Reviewer: “representativeness genotype covariates” to “most representative genotype covariates.”

Response: Corrected.

Reviewer: Break long sentences into shorter sentences for readability.

Response: Sentences were shortened for better readability.

Reviewer: Ensure “hydric deficit” term is used consistently.

Response: Standardized throughout the manuscript.

Reviewer: Clarify subset of nine Tunisian landraces versus broader collections.

Response: Clarified (nine landraces studied from a collection of 223).

Reviewer: Ensure tense consistency (past tense for conducted work).

Response: Tense was standardized.

Reviewer: Correct minor grammar issues: articles, prepositions, plural forms.

Response: Corrected.

Reviewer: Define abbreviations (e.g., ASI, NGP, PH, GYP) in abstract.

Response: Abbreviations have been defined at first mention.

Reviewer: “Mediterranean maize landraces are adapted” to “have adapted”.

Response: Corrected.

Reviewer: Check spelling and consistency of cultivar/landrace names (BK, KAR, MT2, etc.).

Response: Corrected throughout.

Reviewer: Ensure consistent use of “stress conditions” vs. “stress environments.”

Response: Standardized terminology.

Reviewer: Clarify “five checks”—specify whether commercial hybrids or local controls.

Response: Clarified as a mix of commercial hybrids and local controls.

Reviewer: Provide units for all traits measured (e.g., g for biomass, mm for rainfall).

Response: Units for all traits were added.

Reviewer: Ensure consistency in site names (C_22, S_22, D_H, OP) across methods and results.

Response: Corrected and standardized.

Reviewer: Spell out first mention of abbreviations (e.g., DT, DS, GYP, PH, 1000GW, NE, NGP).

Response: All abbreviations were spelled out at first mention.

Reviewer: Correct minor grammar and sentence structure issues: “represents” to “represented” where appropriate.

Response: Corrected.

Reviewer: Ensure consistent landrace names throughout (BK, KAR, MT2, GAF, BIZ1, BIZ2, GAB1, GAB2, MT1).

Response: Corrected throughout.

Reviewer: “showing significant tolerance and adaptability for several stress levels” to “across multiple stress levels”.

Response: Corrected.

Reviewer: “outperformed for most traits” to “outperformed in most traits”.

Response: Corrected.

Reviewer: “Other populations, such as GAF, showed a stable grain yield performance” to “Population GAF showed stable grain yield performance”.

Response: Corrected.

Reviewer: Include units for all traits in tables and figures.

Response: Units were added consistently.

Reviewer: Check tense consistency: past tense for conducted experiments, present for general knowledge.

Response: Corrected.

Reviewer: Remove redundancy in statements like “BK maintained highest performance and surpassed checks in yield.”

Response: Redundancy removed.

Reviewer: Correct minor typographical errors: “cumulated hydric deficit” vs. “cumulative hydric deficit”; “thousand-grain weight” consistently.

Response: Corrected.

Reviewer: Ensure numbers are consistently formatted (e.g., 1000GW vs. 1,000GW).

Response: Corrected.

Reviewer: Standardize decimal points (e.g., 1.21 days vs. 1.2 days).

Response: Standardized.

Reviewer: Make consistent use of % sign with or without a space.

Response: Corrected consistently.

Reviewer: Avoid redundant phrasing: “better yields and flowering synchronization” repeated multiple times.

Response: Redundancy removed.

Reviewer: Shorten excessively long sentences.

Response: Sentences shortened.

Reviewer: Check all citations for correct format and completeness.

Response: Corrected.

Reviewer: Correct minor grammatical issues: “plants may adapt by increasing” to “plants appeared to adapt by increasing.”

Response: Corrected.

Reviewer: Ensure consistent trait naming: “ear number” vs. “number of ears (NE)”, “grain weight” vs. “1000GW.”

Response: Corrected.

Reviewer: Correct small numeric inconsistencies: e.g., percentages of plant height reduction, ASI, GYP between environments.

Response: Corrected.

Reviewer: ‘Sumathi et., 2005’ to ‘Sumathi et al., 2005’.

Response: Corrected.

Reviewer: Replace vague phrases like “significant performance” with specific metrics or values.

Response: Corrected.

Reviewer: Ensure smooth transition between discussion paragraphs for readability.

Response: Transitions improved.

Reviewer: Standardize capitalization in acronyms: e.g., ASI, NGP, PH, GYP, 1000GW throughout the manuscript.

Response: Corrected.

Reviewer: ‘(Hammami et al., 2025b; Troyer’ complete the reference.

Response: Reference completed.

Reviewer: Sharpen take-home message; avoid redundancy with discussion.

Response: Take-home message sharpened and redundancy avoided.

Reviewer: Suggest future research directions with precision: e.g., root traits, molecular markers, controlled trials.

Response: Future research directions added with precision.

Reviewer: Proofread entire manuscript for minor grammatical errors: articles, pluralization, verb tense consistency.

Response: Corrected.

Reviewer: Avoid overly complex or nested sentences; simplify for clarity.

Response: Sentences simplified.

Reviewer: Check spacing around units, numbers, and symbols.

Response: Corrected.

In summary, all your concerns, both major and minor, were addressed. The manuscript has been thoroughly revised to improve clarity, scientific accuracy, and breeding relevance.

We are grateful for your valuable feedback, which has greatly contributed to strengthening the manuscript.

Sincerely,

Hammami Mohamed Dhia Eddine

Attachment Submitted filename: Response_to_Reviewer.docx

# Editor report: Decision Letter 2

**DOI:** 10.1371/journal.pone.0338577.r005

**Authors:** Mehdi Rahimi

25 Nov 2025

Genotypic variability of Tunisian maize landraces: A Valuable Genetic Resource to mitigate Drought and heat stress in the Mediterranean basin

PONE-D-25-21948R2

Dear Dr. Hammami,

We’re pleased to inform you that your manuscript has been judged scientifically suitable for publication and will be formally accepted for publication once it meets all outstanding technical requirements.

Within one week, you’ll receive an e-mail detailing the required amendments. When these have been addressed, you’ll receive a formal acceptance letter and your manuscript will be scheduled for publication.

An invoice will be generated when your article is formally accepted. Please note, if your institution has a publishing partnership with PLOS and your article meets the relevant criteria, all or part of your publication costs will be covered. Please make sure your user information is up-to-date by logging into Editorial Manager at Editorial Manager®  and clicking the ‘Update My Information' link at the top of the page. For questions related to billing, please contact billing support .

If your institution or institutions have a press office, please notify them about your upcoming paper to help maximize its impact. If they’ll be preparing press materials, please inform our press team as soon as possible -- no later than 48 hours after receiving the formal acceptance. Your manuscript will remain under strict press embargo until 2 pm Eastern Time on the date of publication. For more information, please contact onepress@plos.org.

Kind regards,

Mehdi Rahimi, Ph.D.

Academic Editor

PLOS ONE

Additional Editor Comments (optional):

Reviewers' comments:

# Editor report: Acceptance letter

**DOI:** 10.1371/journal.pone.0338577.r006

**Authors:** Mehdi Rahimi

PONE-D-25-21948R2

PLOS ONE

Dear Dr. Hammami,

I'm pleased to inform you that your manuscript has been deemed suitable for publication in PLOS ONE. Congratulations! Your manuscript is now being handed over to our production team.

At this stage, our production department will prepare your paper for publication. This includes ensuring the following:

* All references, tables, and figures are properly cited

* All relevant supporting information is included in the manuscript submission,

* There are no issues that prevent the paper from being properly typeset

You will receive further instructions from the production team, including instructions on how to review your proof when it is ready. Please keep in mind that we are working through a large volume of accepted articles, so please give us a few days to review your paper and let you know the next and final steps.

Lastly, if your institution or institutions have a press office, please let them know about your upcoming paper now to help maximize its impact. If they'll be preparing press materials, please inform our press team within the next 48 hours. Your manuscript will remain under strict press embargo until 2 pm Eastern Time on the date of publication. For more information, please contact onepress@plos.org.

You will receive an invoice from PLOS for your publication fee after your manuscript has reached the completed accept phase. If you receive an email requesting payment before acceptance or for any other service, this may be a phishing scheme. Learn how to identify phishing emails and protect your accounts at https://explore.plos.org/phishing.

If we can help with anything else, please email us at customercare@plos.org.

Thank you for submitting your work to PLOS ONE and supporting open access.

Kind regards,

PLOS ONE Editorial Office Staff

on behalf of

Associate Prof. Mehdi Rahimi

Academic Editor

PLOS ONE
